# Supplementary material for: Sustainable conversion of alkaline nitrate to ammonia at activities greater than 2 A cm−2
Source: Nat Commun. 2024 Feb 10;15:1264. doi: 10.1038/s41467-024-45534-2 (PMC10858923; doi:10.1038/s41467-024-45534-2)
Supplement: Supplementary file 1 — Supplementary Information [file 41467_2024_45534_MOESM1_ESM.pdf]

Supplementary Information

Sustainable conversion of alkaline nitrate to ammonia at activities greater than 2 A cm^{-2}

Wanru Liao[1,+], Jun Wang[1,+], Ganghai Ni[1], Kang Liu[1], Changxu Liu[2], Shanyong Chen[3], Qiyou Wang[1], Yingkang Chen[1], Tao Luo[1], Xiqing Wang[1], Yanqiu Wang[3], Wenzhang Li[3], Ting-Shan Chan[4], Chao Ma[5], Hongmei Li[1], Ying Liang[6], Weizhen Liu[7], Junwei Fu[1,*], Beidou Xi[8,*], Min Liu[1,*]

[1] Hunan Joint International Research Center for Carbon Dioxide Resource Utilization, State Key Laboratory of Powder Metallurgy, School of Physics, Central South University, Changsha 410083, P. R. China.

[2] Centre for Metamaterial Research & Innovation, Department of Engineering, University of Exeter, Exeter EX4 4QF, UK.

[3] School of Chemistry and Chemical Engineering, Central South University, Changsha 410083, P. R. China.

[4] National Synchrotron Radiation Research Center, Hsinchu 300092, Taiwan.

[5] College of Materials Science and Engineering, Hunan University, Changsha 410082, P. R. China.

[6] College of Food Science and Engineering, Central South University of Forestry and Technology, Changsha 410004, P. R. China

[7] School of Environment and Energy, Guangdong Provincial Key Laboratory of Solid Wastes Pollution Control and Recycling, South China University of Technology, Guangzhou 510006, P. R. China.

[8] State Key Laboratory of Environmental Criteria and Risk Assessment, Chinese Research Academy of Environmental Sciences, Beijing 100012, P. R. China.

[+] These authors contributed equally to this work.

Corresponding authors: fujunwei@csu.edu.cn, xibd@craes.org.cn, minliu@csu.edu.cn

Experimental Section:

Calculation of the Faradaic efficiency and the yield rate for NH_3.

The NH_3 Faradaic efficiency for NO_3RR could be calculated as follows:

$$FE(NH_3) = (n \times F \times c_{NH3} \times V) / (M \times Q) \quad (1)$$

The NH_3 yield rate for NO_3RR could be calculated as follows:

$$\text{Yield rate}(NH_3) = (c_{NH3} \times V) / (t \times A) \quad (2)$$

Where n is the electron-transfer number (for 1 mol NH_3, it was 8), F was the Faradaic constant (96,485 C mol^{-1}), c_{NH3} was the measured NH_3 concentration (μg mL^{-1}), V was the volume of the cathode electrolyte (32 mL), M was the relative molecular mass of products (17), Q was the applied overall coulomb quantity (C), A was the area of the catalyst electrode (1 cm^2), and t was the reaction time (1 h).

ECSA analysis.

The electrochemical double-layer capacitance (C_{dl}) of the materials was tested to determine their electrochemical surface area (ECSA) using the cyclic voltammetry (CV) in non-faradic regions with diverse scan rates ranging from 10 to 50 mV s^{-1} between 0.975 and 1.025 V vs. RHE. The plotted current density (difference between the anode current density and cathode current density at 1.0 V vs. RHE) against scan rate has shown a linear relationship and its slope was twice the C_{dl}. The ECSA was determined by:

$$ECSA = C_{dl} / C_s \quad (3)$$

The surface-area-normalized activity of NH_3 could be calculated as below:

$$\text{Yield rate}_{ECSA}(NH_3) = (c_{NH3} \times V) / (t \times ECSA) \quad (4)$$

where C_s was the specific capacitance of the sample, a general specific capacitance of $C_s = 0.040$ mF cm^{-2} was used in this study. c_{NH3} was the measured NH_3 concentration (μg mL^{-1}), V was the volume of the cathode electrolyte (32 mL), and t was the reaction time (1 h).

Calculation of nitrate conversion and product selectivity.

The conversion of NO_3^- could be calculated as below:

$$C(NO_3^-)\% = (C_0(NO_3^--N) - C_t(NO_3^--N)) / C_0(NO_3^--N) \times 100\% \quad (5)$$

The conversion of NO_3^- could be calculated as below:

$$S(NO_2^-)\% = C_t(NO_2^--N) / (C_0(NO_3^--N) - C_t(NO_3^--N)) \times 100\% \quad (6)$$

$$S(NH_4^+)\% = C_t(NH_4^+-N) / (C_0(NO_3^--N) - C_t(NO_3^--N)) \times 100\% \quad (7)$$

Where $C_0(NO_3^--N$ (mg L^{-1}) and $C_t(NO_3^--N)$ (mg L^{-1}) were the initial nitrate concentration and the nitrate concentration at different electrolysis time, respectively. $C_t(NO_2^--N)$ and $C_t(NH_4^+-N)$ were the concentrations of nitrite and ammoniums at different electrolysis time.

Characterizations.

X-ray diffraction (XRD) data were recorded over an X'Pert3 Powder diffractometer using Cu Kα radiation (45 kV, 40 mA). The morphology images were studied by scanning electron microscopes (SEM, F EI Helios Nanolab 600i). transmission electron microscopy (TEM) images were obtained from FEI Tecnai G 2 F20 field emission transmission electron microscope operated at 200 kV, equipping with energy dispersive X-ray spectroscopy (EDS) mapping. The aberration-corrected high-angle annular dark-field scanning transmission electron microscopy (AC-HAADF-STEM) images were obtained on the JEOL ARM200F microscope with a probe-forming spherical aberration corrector. The chemical state and composition of the samples were characterized using X-ray photoelectron spectroscopy (XPS, Thermo Scientific K-Alpha) with an Al Kα ($h\nu$ =1486.6 eV) monochrome. All binding energies were referenced to the C 1*s* peak (284.6 eV) of the surface adventitious carbon. The X-ray absorption spectroscopy (XAS) spectra were measured on a hard X-ray spectrometer at the TLS 01C1 and TLS 16A1 beamlines of the National Synchrotron Radiation Research Center (NSRRC, Taiwan) in the fluorescence mode. The corresponding XAFS data were analyzed through the standard procedures using the Ifeffit package. The XAS data were analyzed using the software package Athena. Ammonia, nitrate, and nitrite were detected by ultraviolet-

visible diffuse reflectance spectroscopy (UV-vis) using a UV-2600 spectrophotometer (Shimadzu).

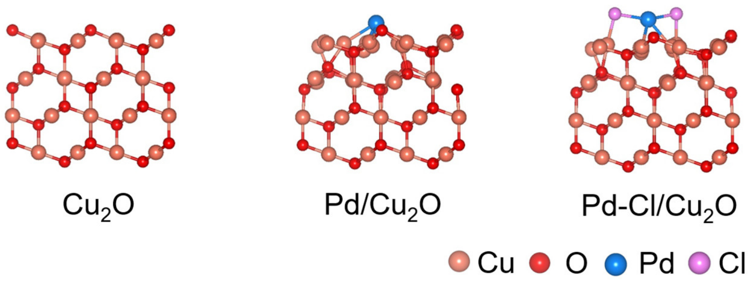

Supplementary Fig. 1 | Cu_2O, Pd/Cu_2O and Pd-Cl/Cu_2O models.

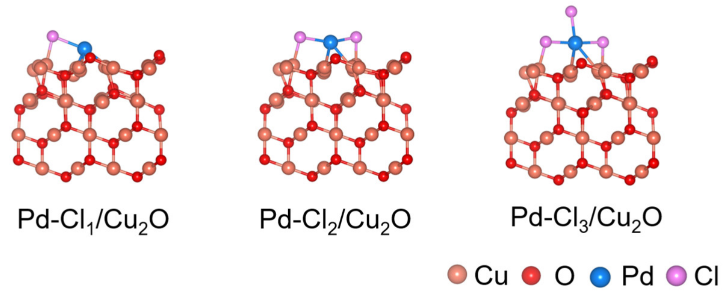

Supplementary Fig. 2 | The models of Pd-Cl/Cu_2O with different Cl numbers.

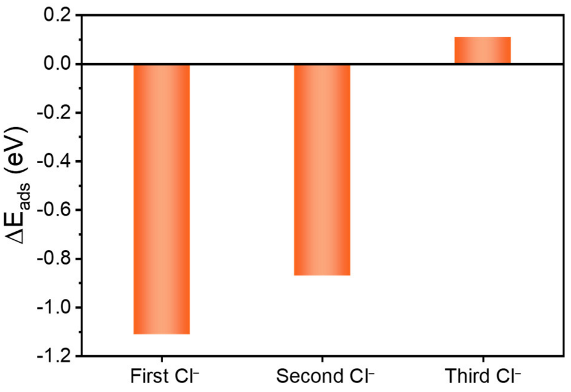

Supplementary Fig. 3 | The adsorbed energy of the first, second, and third Cl^- on the $Pd\text{-}Cl_{(n-1)}/Cu_2O$ configuration (n=1, 2, 3).

Notes: Choosing a model should indeed be based on its energetically favorable system. Thus, we calculated the adsorbed energy of gradually increased each Cl^- in the $Pd\text{-}Cl_{(n-1)}/Cu_2O$ (n= 1, 2, 3) structure, to select the most stable $Pd\text{-}Cl/Cu_2O$ model. The adsorbed energy of Cl^- can be evaluated according to the formula of $\Delta E_{ads} = E_{Pd\text{-}Cln/Cu2O} - E_{Pd\text{-}Cl(n-1)/Cu2O} - E_{Cl^-}$ (n = 1, 2, 3). As shown in Supplementary Fig. 3, the energy of the first, second, and third Cl^- adsorbed on the $Pd\text{-}Cl_{(n-1)}/Cu_2O$ configuration are -1.11, -0.87, and 0.11 eV, respectively (Supplementary Fig. 2). Therefore, the two Cl^- adsorbed on Pd/Cu_2O ($Pd\text{-}Cl_2/Cu_2O$) is the most stable, and we chose this model to study its reaction behavior.

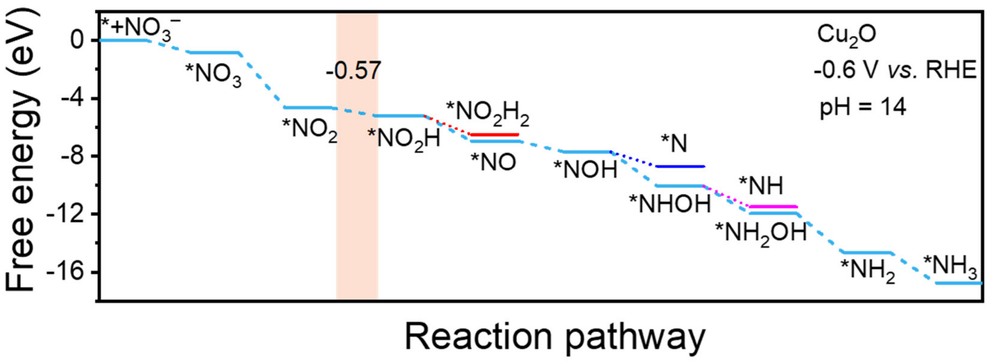

Supplementary Fig. 4 | Gibbs free energy diagram of various intermediates generated during NO_3RR over Cu_2O at the potential of -0.6 V vs. RHE for pH=14.

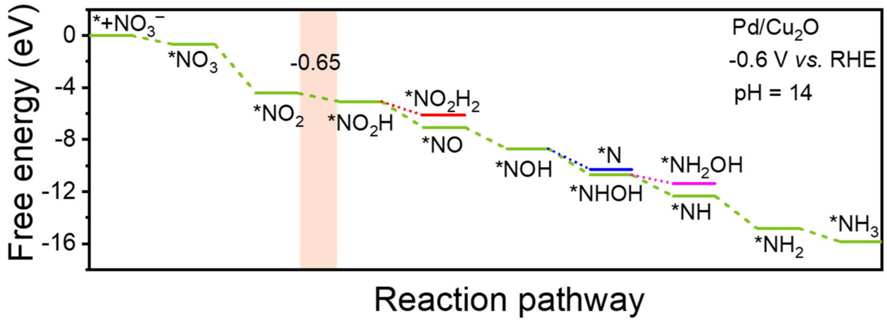

Supplementary Fig. 5 | Gibbs free energy diagram of various intermediates generated during NO_3RR over Pd/Cu_2O at the potential of -0.6 V vs. RHE for pH=14.

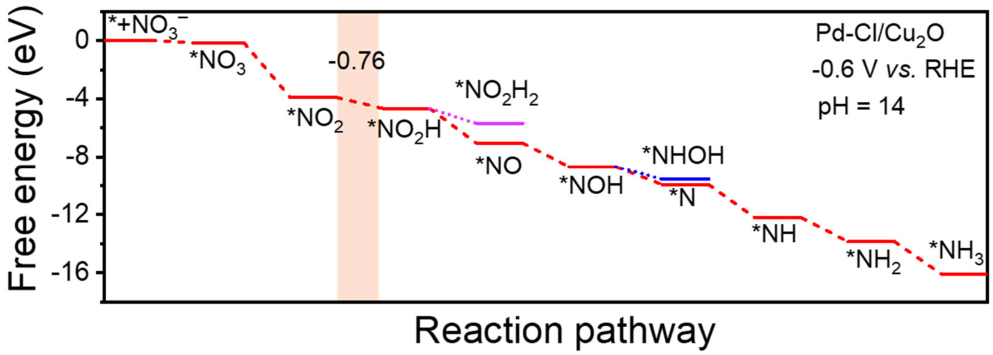

Supplementary Fig. 6 | Gibbs free energy diagram of various intermediates generated during NO_3RR over Pd-Cl/Cu_2O at the potential of -0.6 V vs. RHE for pH=14.

Notes: We calculated the free energy changes of the NO_3RR over Cu_2O, Pd/Cu_2O and Pd-Cl/Cu_2O at the potential of -0.6 V vs. RHE for pH=14. Since NO_3RR is highly complex with multiple possible branches, we chose the optimal pathway after comparing alternative pathways. For Cu_2O (Supplementary Fig. 4), the energy is more significantly downhill in *NO_2H → *NO, *NOH → *NHOH and *NHOH → *NH_2OH steps than that in *NO_2H → *NO_2H_2, *NOH → *N and *NHOH → *NH steps, respectively. Therefore, Cu_2O is more likely to undergo the pathway of *NO_3 → *NO_2 →*NO_2H → *NO → *NOH → *NHOH → *NH_2OH → *NH_2 →*NH_3. Similarly, Pd/Cu_2O (Supplementary Fig. 5) is more inclined to follow the pathway of *NO_3 → *NO_2 →*NO_2H → *NO → *NOH → *NHOH → *NH → *NH_2 →*NH_3, and Pd-Cl/Cu_2O (Supplementary Fig. 6) tends to follow the pathway of *NO_3 → *NO_2 →*NO_2H → *NO → *NOH → *N → *NH → *NH_2 →*NH_3.

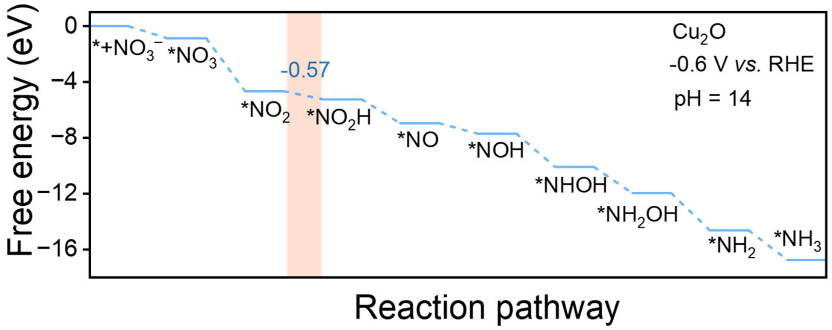

Supplementary Fig. 7 | Gibbs free energy diagram of various intermediates generated during NO_3RR over Cu_2O at the potential of -0.6 V vs. RHE for pH=14.

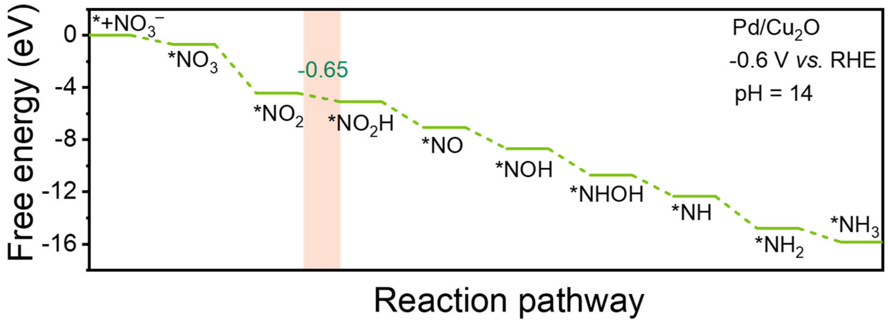

Supplementary Fig. 8 | Gibbs free energy diagram of various intermediates generated during NO_3RR over Pd/Cu_2O at the potential of -0.6 V vs. RHE for pH=14.

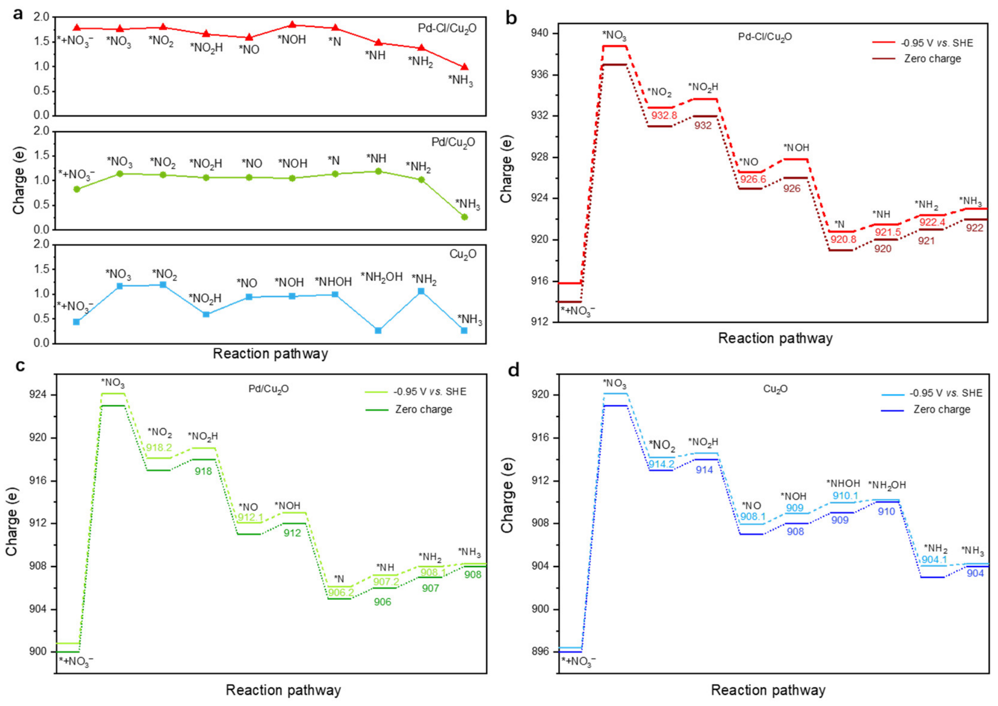

Supplementary Fig. 9 | a, The charge vibration of each intermediate on the catalysts between the potentials of zero charge and -0.95 V vs. SHE. **b**, The charge of each intermediate on Pd-Cl/Cu_2O at the potentials of zero charge and -0.95 V vs. SHE. **c**, The charge of each intermediate on Pd/Cu_2O at the potentials of zero charge and -0.95 V vs. SHE. **d**, The charge of each intermediate on Cu_2O at the potentials of zero charge and -0.95 V vs. SHE.

Notes: To determine the type of proton-electron transfer in the nitrate reduction process, the nitrate reduction pathways on Cu_2O, Pd/Cu_2O and Pd-Cl/Cu_2O models in our research system (pH=14) have been calculated. The theoretical potential for nitrate reduction to ammonia was -0.12 V vs. SHE. According to the calculation of Nernst's equation (E_RHE = E_SHE + 0.0591×pH), the corresponding theoretical potential was -0.12 V vs. RHE at pH=0. Thus, under the potentials of -0.12 V vs. RHE, the corresponding reaction potential at pH=14 was -0.95 V vs. SHE. We calculate the reaction pathway on the three catalysts at the potential of -0.95 V vs. SHE and compare the charge vibration of each intermediate on the three catalysts between the potentials of zero charge and -0.95 V vs. SHE.

Take Pd-Cl/Cu_2O as an example. Before adsorbing nitrate (Supplementary Fig. 9a), the charge number of Pd-Cl/Cu_2O at -0.95 V vs. SHE is more than that of the zero charge potential (1.78 *e*),

indicating that Pd-Cl/Cu_2O has obtained electrons at the potential of -0.95 V vs. SHE. That is, the electrons are first transferred to Pd-Cl/Cu_2O. After adsorbing nitrate (*NO_3), the charge number on Pd-Cl/Cu_2O at -0.95 V vs. SHE is still more than that of the zero charge potential (1.76 *e*), which demonstrates many electrons transfer to the reaction system under potential driving. As shown in Supplementary Fig. 9b, in the hydrogenation process of *NO_2 to *NO_2H (*NO_2 + e^- + H^+ → *NO_2H), the charge number on Pd-Cl/Cu_2O with absorbed NO_2H at the potential of zero charge (932 *e*) is smaller than that of the system where the catalyst adsorbs NO_2 (*NO_2, 932.8 *e*) at the potential of -0.95 V vs. SHE (H^+ has not yet participated in the reaction). This result proves that electrons are transferred to Pd-Cl/Cu_2O under the action of potential before H^+ coupling. There are similar transfer phenomena in the following nitrate intermediate hydrogenation process on Pd-Cl/Cu_2O (*NO → *NOH, *N → *NH, *NH→ *NH_2, *NH_2→*NH_3). The phenomena in which electrons are transferred to the catalyst before H^+ coupling also occur on Cu_2O and Pd/Cu_2O. Thus, the three catalysts undergo a sequential electron–proton transfer process in the nitrate reduction pathway (Supplementary Fig. 9).

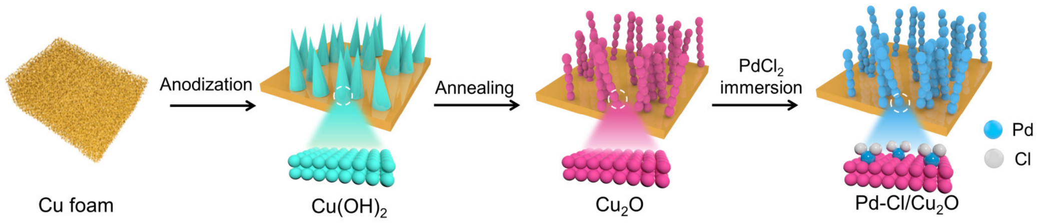

Supplementary Fig. 10 | Schematic illustration for the synthetic process of Pd-Cl/Cu_2O.

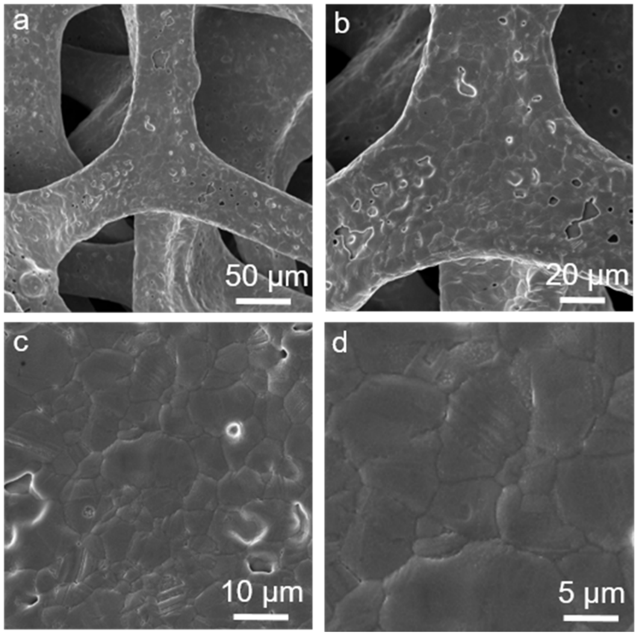

Supplementary Fig. 11 | SEM images of Cu foam with the scale bars of (a) 50 μm, (b) 20 μm, (c) 10 μm, and (d) 5 μm.

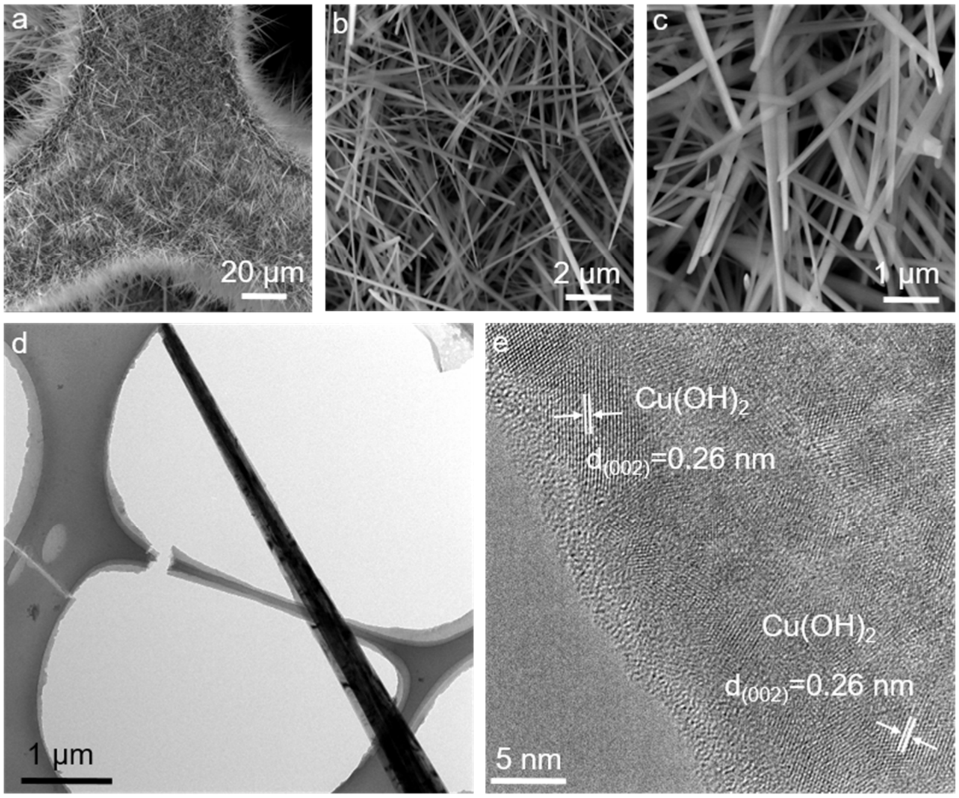

Supplementary Fig. 12 | (a-c) SEM, (d) TEM, and (e) HRTEM images of $Cu(OH)_2$ nanoneedle.

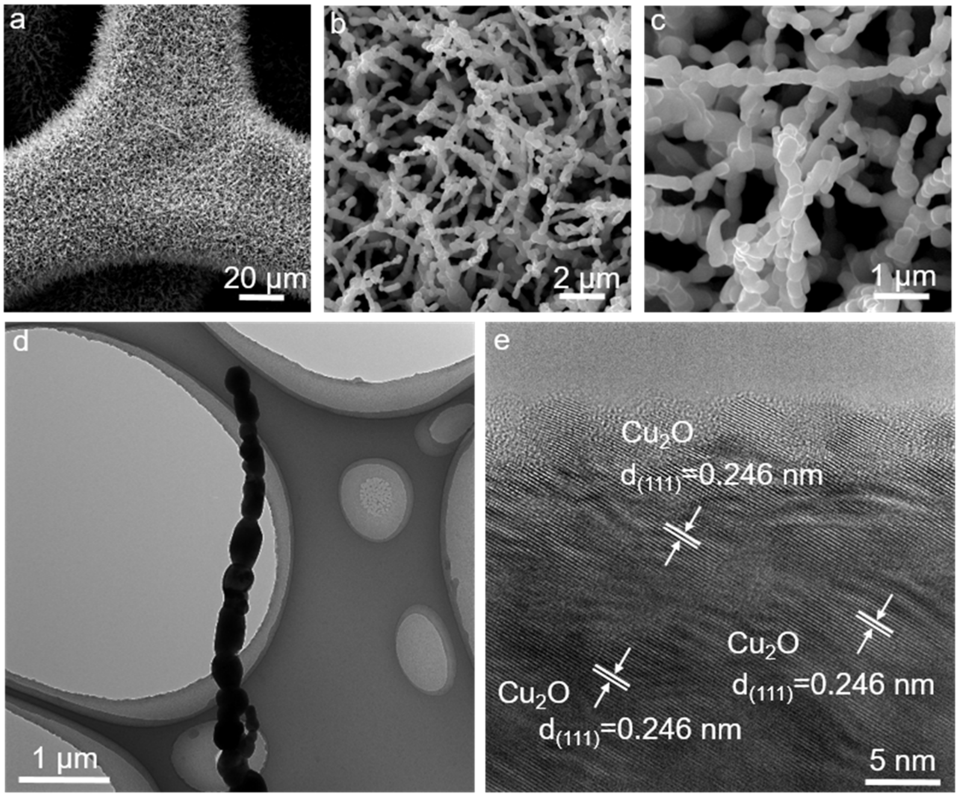

Supplementary Fig. 13 | (a-c) SEM, (d) TEM, and (e) HRTEM images of lotus-like Cu_2O.

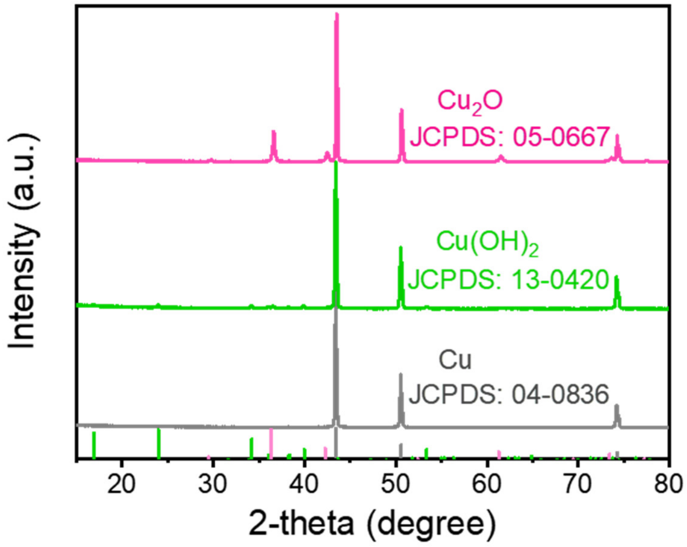

Supplementary Fig. 14 | XRD patterns of catalysts.

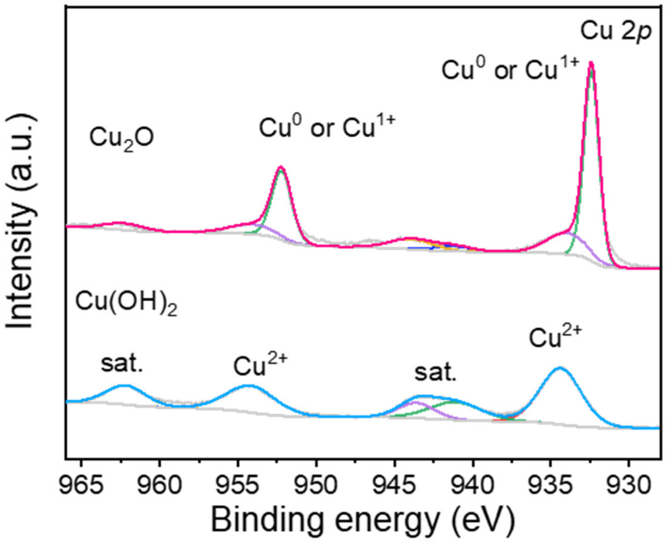

Supplementary Fig. 15 | Cu 2*p* XPS spectra of catalysts.

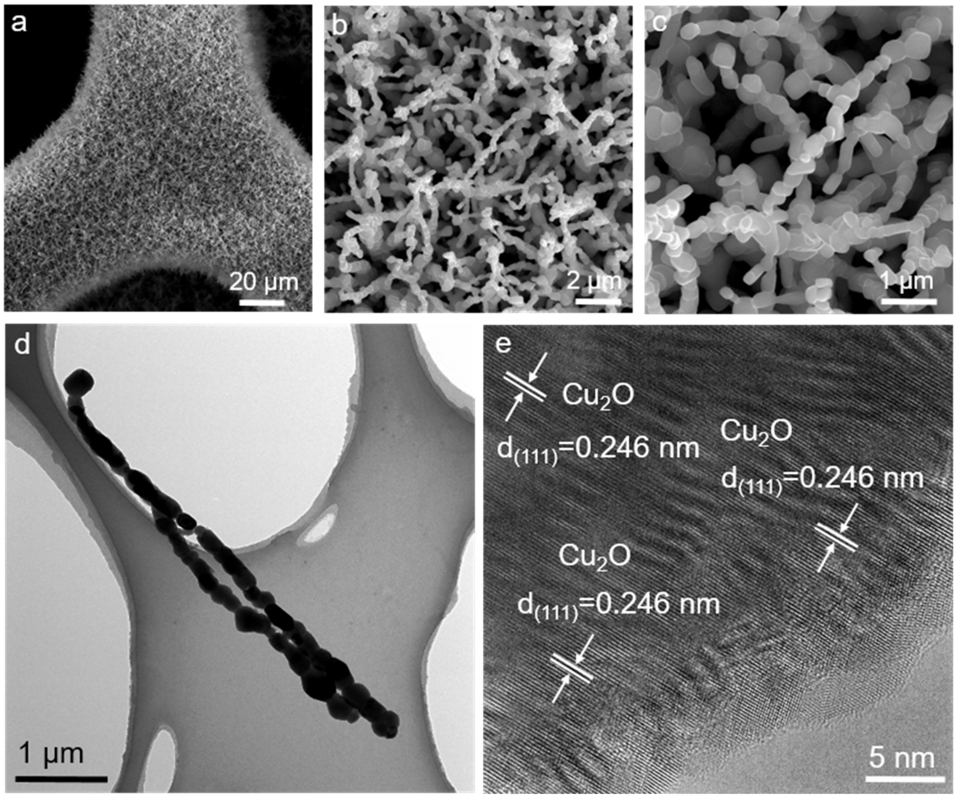

Supplementary Fig. 16 | (a-c) SEM, (d) TEM, and (e) HRTEM images of Pd/Cu_2O.

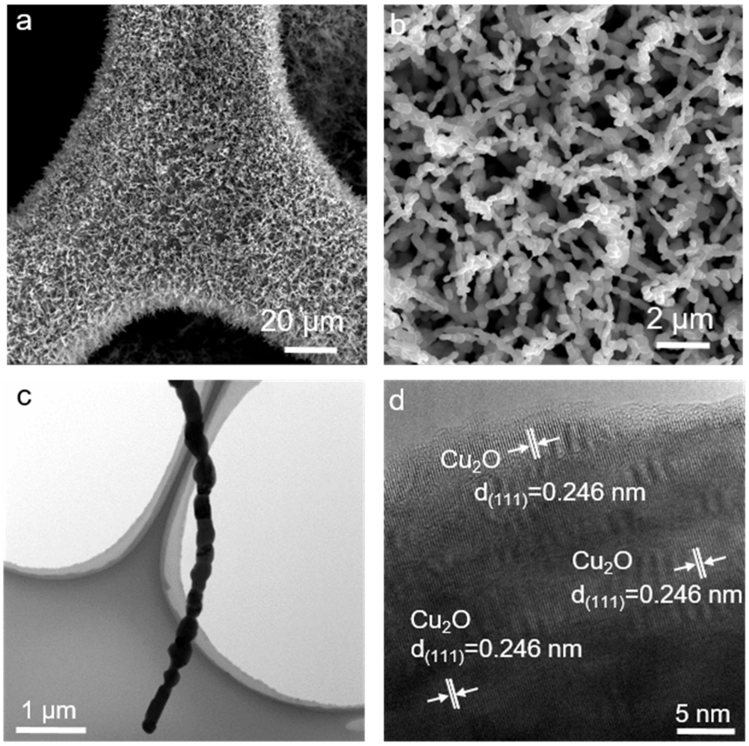

Supplementary Fig. 17 | (a-b) SEM, (c) TEM, and (d) HRTEM images of Pd-Cl/Cu_2O.

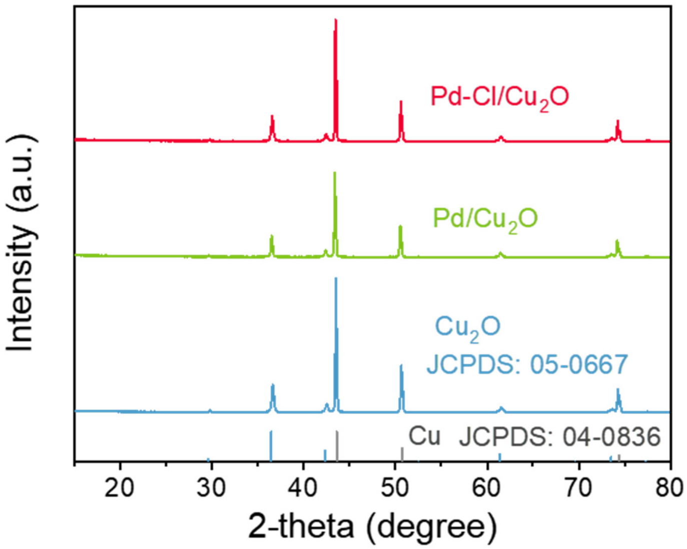

Supplementary Fig. 18 | XRD patterns of catalysts.

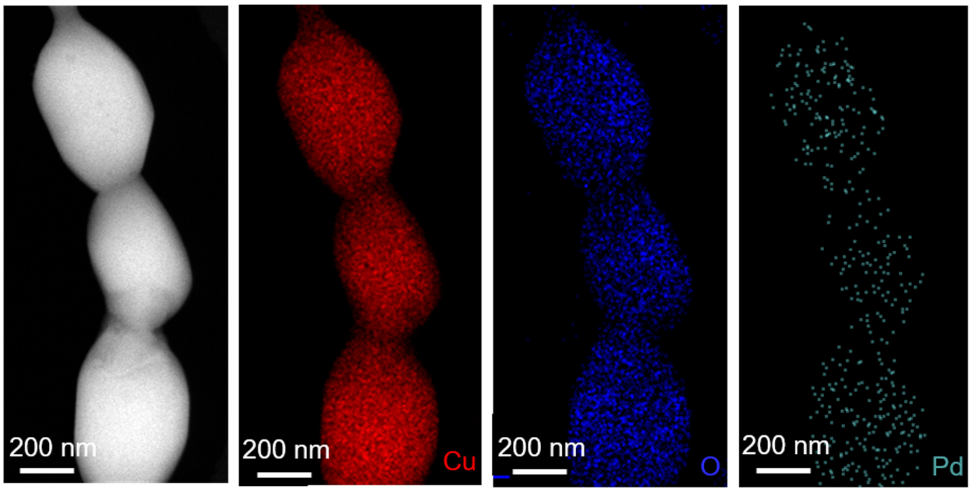

Supplementary Fig. 19 | EDX mapping of Pd/Cu_2O.

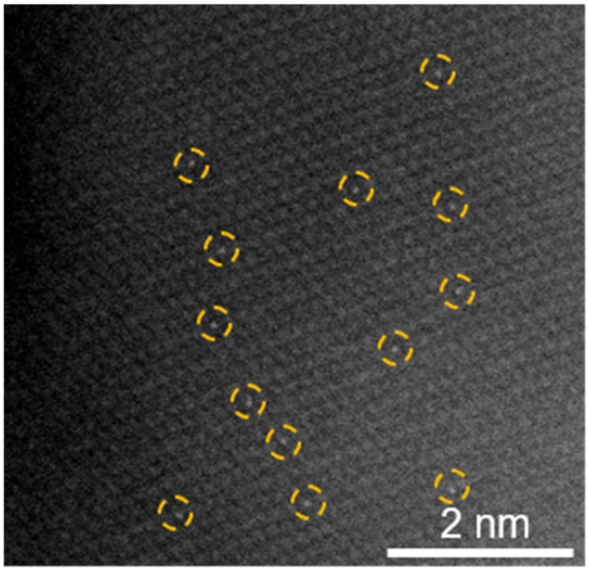

Supplementary Fig. 20 | AC-HAADF-STEM image of Pd/Cu_2O.

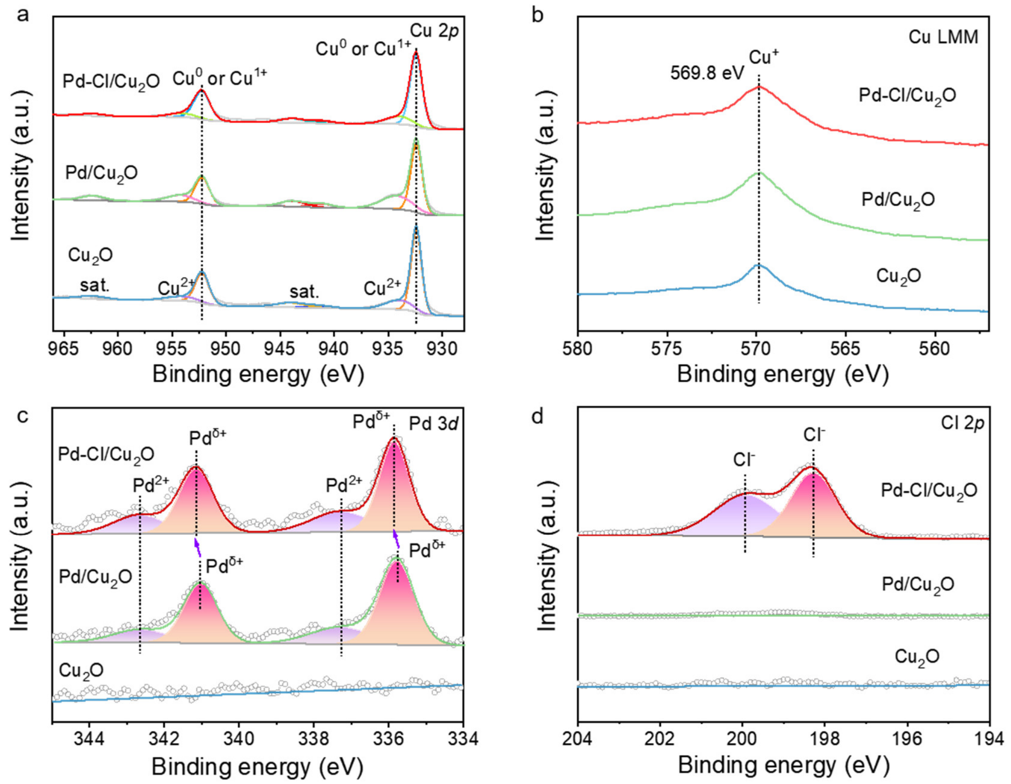

Supplementary Fig. 21 | (a) Cu 2*p* XPS spectra, (b) Cu LMM Auger spectra, (c) Pd 3*d*, and (d) Cl 2*p* XPS spectra of samples.

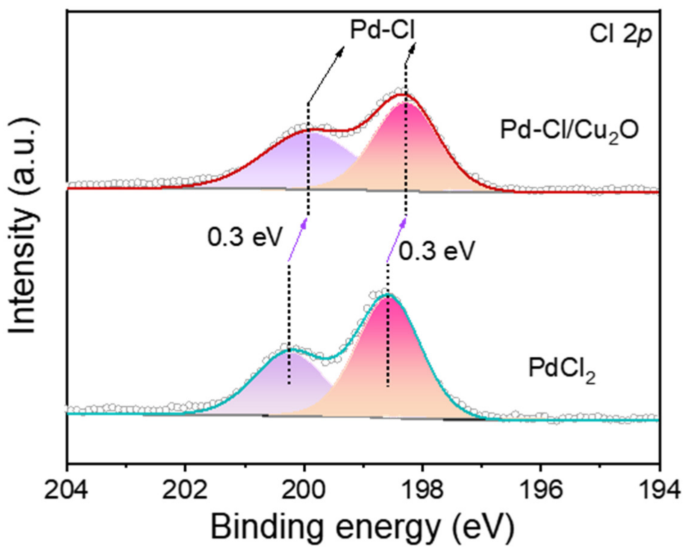

Supplementary Fig. 22 | Cl 2*p* XPS spectra of catalysts.

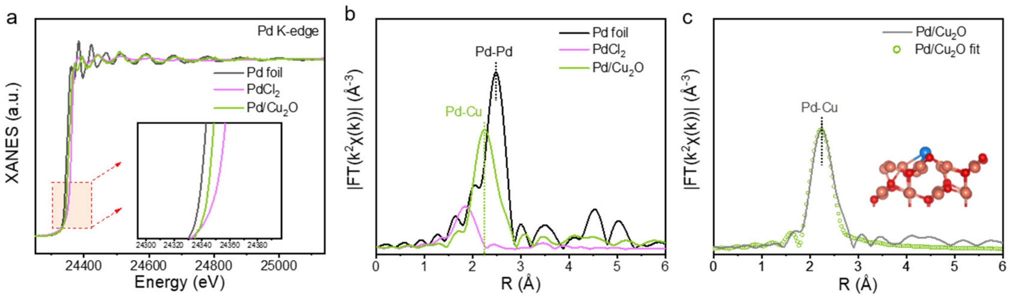

Supplementary Fig. 23 | (a) Pd K-edge XANES spectra, and (b) FT k^2-weighted EXAFS spectra of Pd/Cu_2O and reference samples. (c) The fitting EXAFS spectra of Pd/Cu_2O. Inset: fitting model.

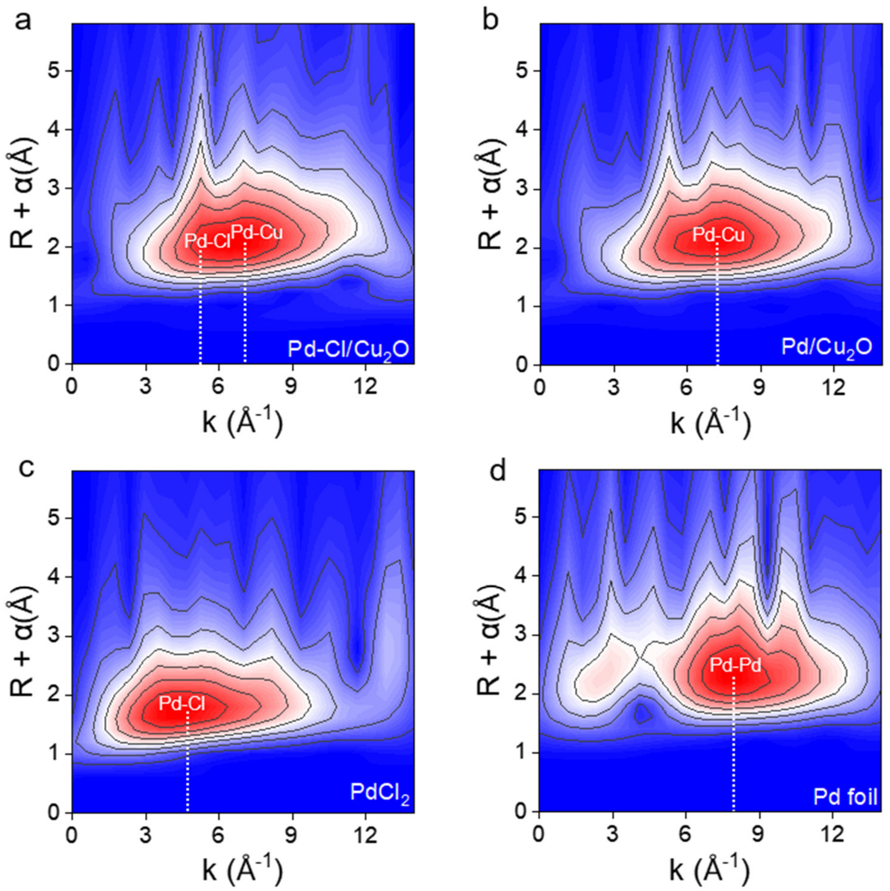

Supplementary Fig. 24 | (a-d) Wavelet transform (WT) k^2-weighted EXAFS contour plots of catalysts.

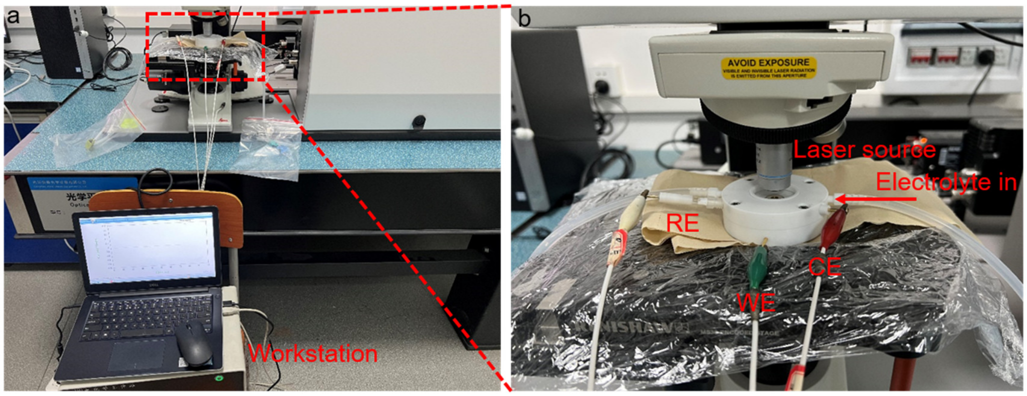

Supplementary Fig. 25 | (a) Digital image of in situ Raman device. (b) Corresponding partially enlarged image.

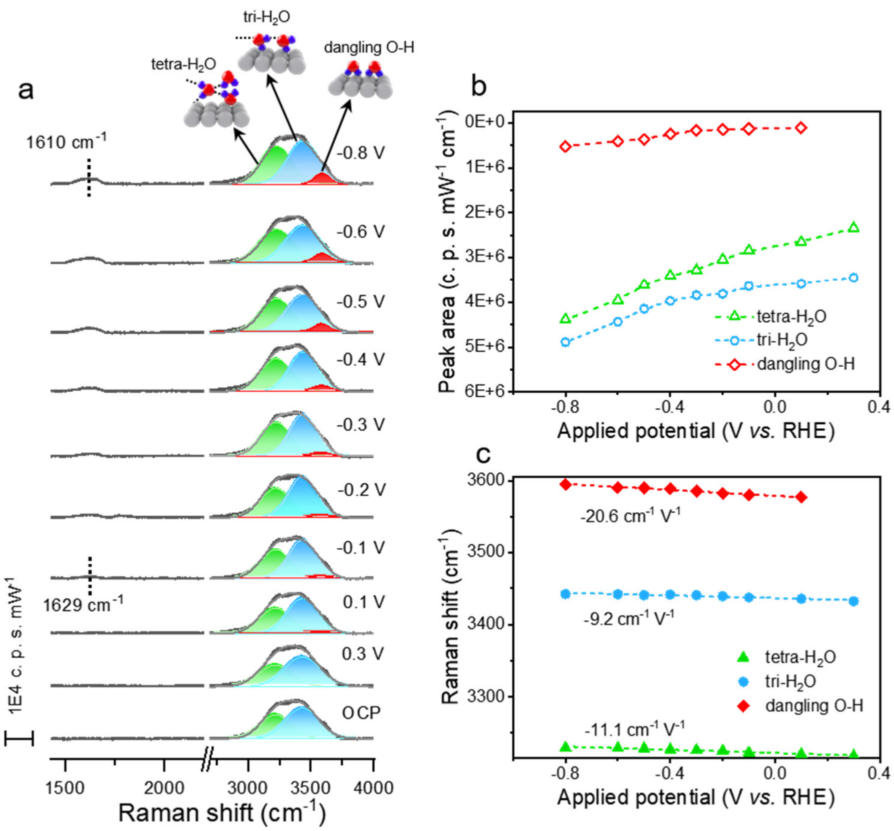

Supplementary Fig. 26 | (a) In situ Raman spectra of Pd/Cu_2O, corresponding (b) peak area and (c) Raman shift of various interfacial water structures.

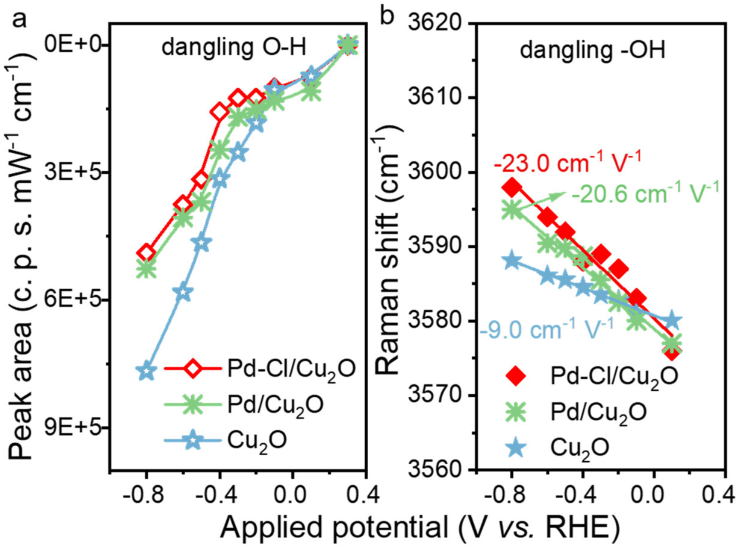

Supplementary Fig. 27 | (a) Peak area and (b) Raman shift of dangling O-H water conducted from in situ Raman spectra of the three catalysts.

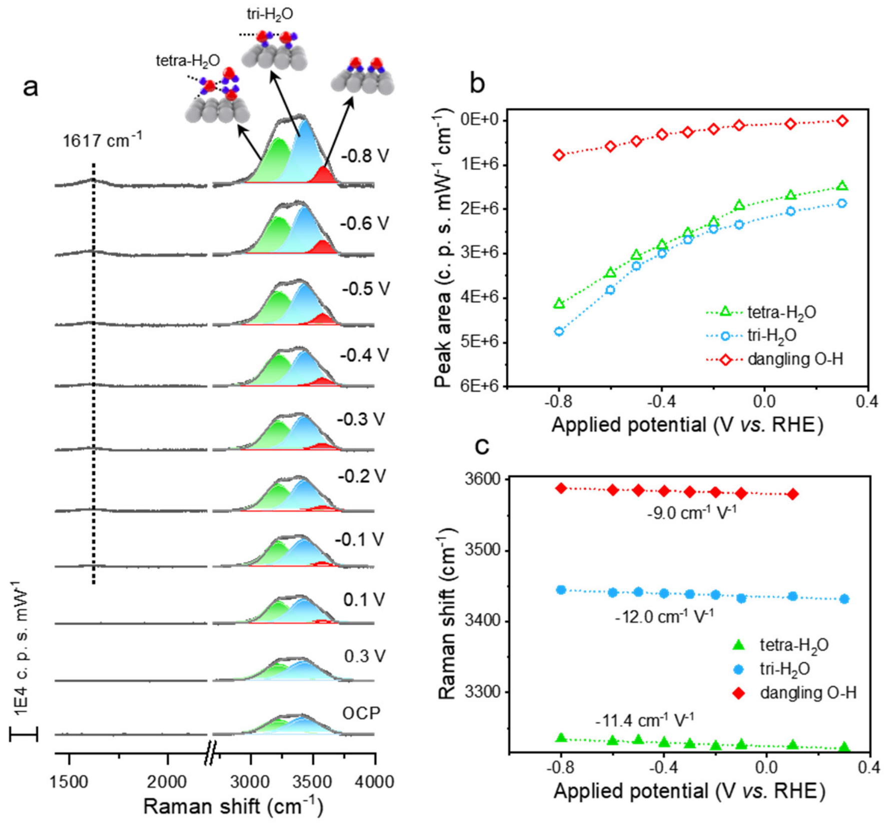

Supplementary Fig. 28 | (a) In situ Raman spectra of Cu_2O, corresponding (b) peak area and (c) Raman shift of various interfacial water structures.

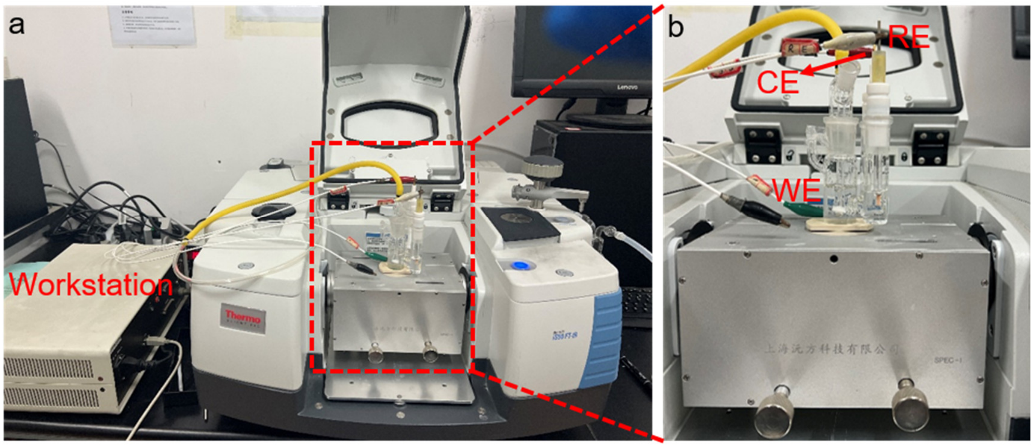

Supplementary Fig. 29 | (a) Digital image of in situ ATR-IR device. (b) Corresponding partially enlarged image.

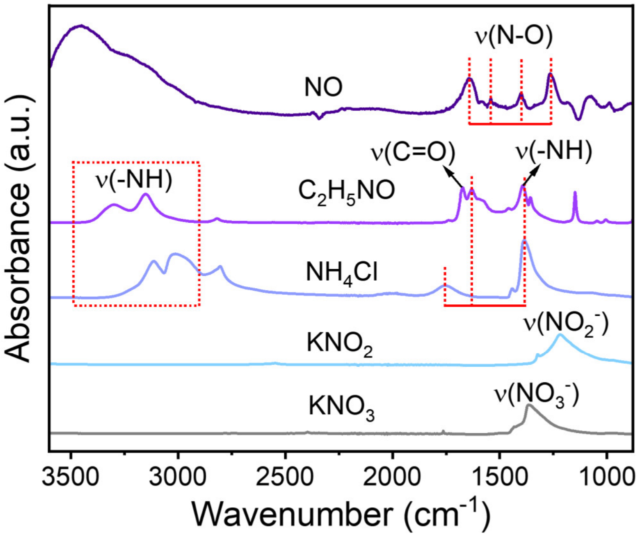

Supplementary Fig. 30 | The IR spectra of the KNO_3, KNO_2, NH_4Cl, C_2H_5NO and NO standard samples.

Notes: We tested the IR spectra of the KNO_3, KNO_2, NH_4Cl, CH_3CONH_2 and NO standard samples. According to the results in Supplementary Fig. 30, the characteristic peaks in the in situ ATR-IR spectra (Figs. 4f-g and Supplementary Fig. 31) have been assigned.

Specifically, the absorption bands at 1362 and 1216 cm^{-1} are assigned to $\nu(NO_3^-)$ and $\nu(NO_2^-)$, respectively, according to the results of standard KNO_3 and KNO_2 powders. Based on the spectra of NH_4Cl and C_2H_5NO, we have identified the characteristic vibration of N-H bond, ranging from 2900-3500 cm^{-1} (red dashed region) and 1380-1760 cm^{-1} (red dotted lines). The peaks at 1674 cm^{-1} in the spectra of C_2H_5NO can be indexed to ν(C=O). In addition, standard NO gas exhibits the characteristic bands at 1260, 1400, 1540, and 1640 cm^{-1}, in accordance with the previously reported works (J. Catal. 237, 393–404, (2006); Langes Handbook of Chemistry.). Besides, the obvious negative peaks at 1114 cm^{-1} in the in situ ATR-IR spectra (Figs. 4f-g and Supplementary Fig. 31) can be ascribed to the Si-O signal (Adv. Mater. Lett. 7, 480-484 (2016); Mater. Sci. Eng. B 105, 209-213 (2003).), which is derived from the reduction of surface SiO_2 on the Si semi-cylindrical prism substrate under the applied potentials.

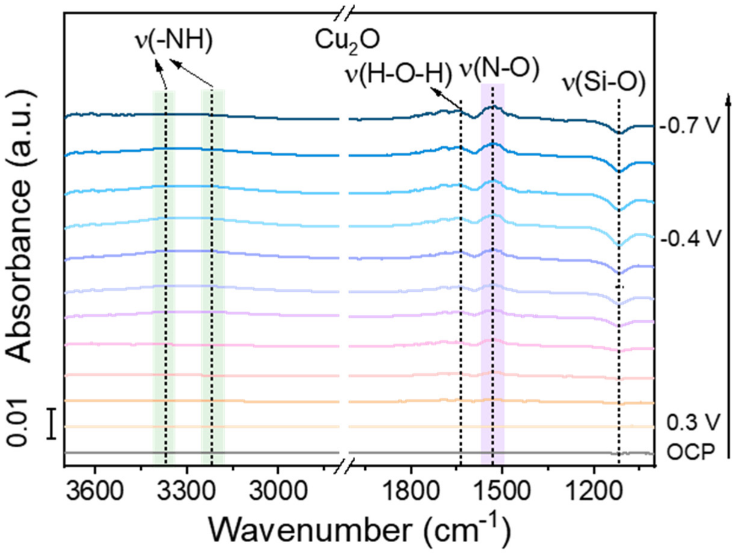

Supplementary Fig. 31 | In situ ATR-IR spectra of Cu_2O catalysts.

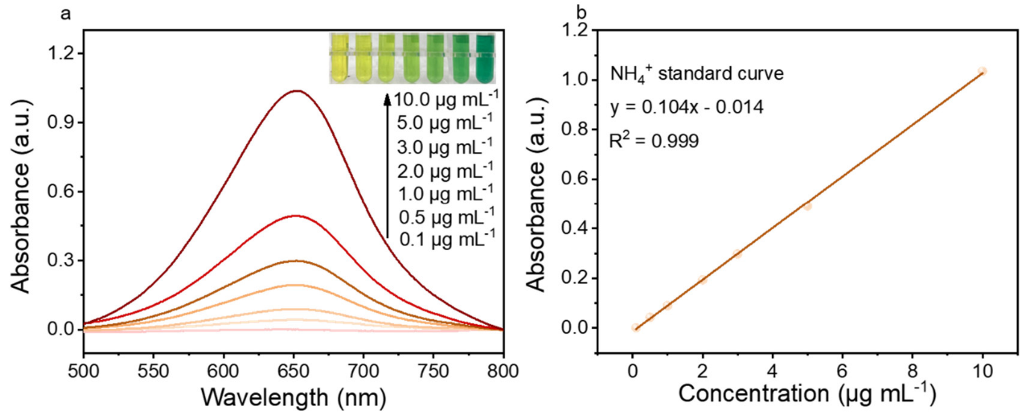

Supplementary Fig. 32 | (a) UV-vis spectra of NH_4^+ reference with various concentrations. Insert picture is the digital image of NH_4^+ reference with various concentrations tested by indophenol blue method. (b) The calibration curve of NH_4^+ reference by UV-vis spectra at room temperature.

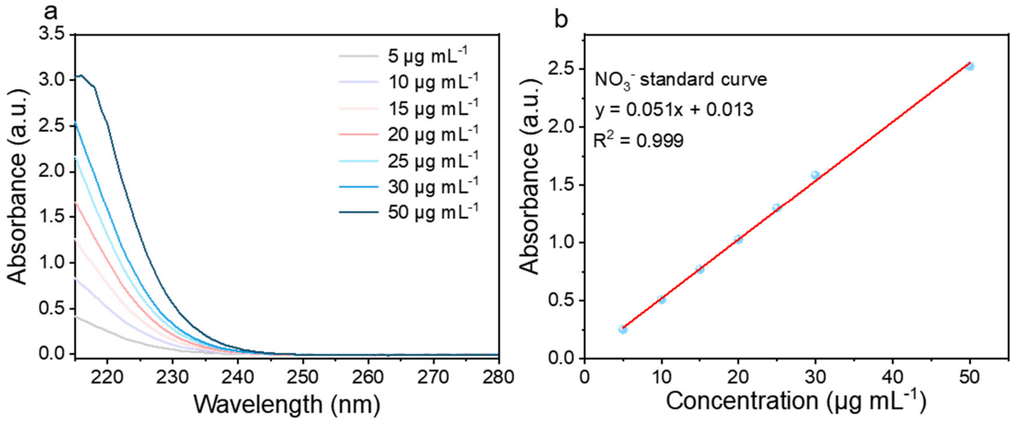

Supplementary Fig. 33 | (a) UV-vis spectra of NO_3^- reference with various concentrations. (b) The calibration curve of NO_3^- reference by UV-vis spectra at room temperature.

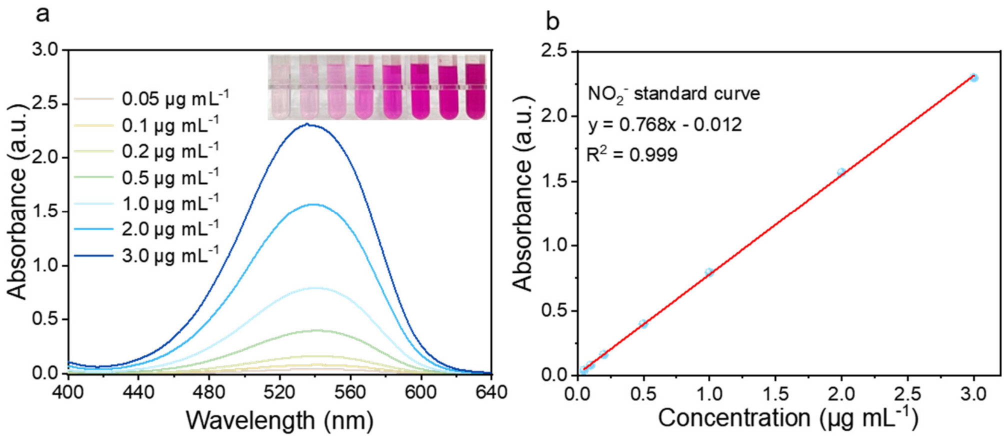

Supplementary Fig. 34 | (a) UV-vis spectra of NO_2^- reference with various concentrations. Insert picture is the digital image of NO_2^- reference with various concentrations tested by chromogenic reaction. (b) The calibration curve of NO_2^- reference by UV-vis spectra at room temperature.

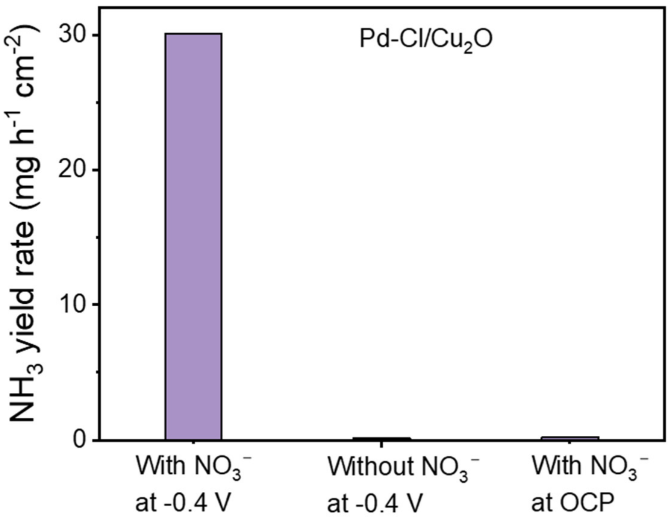

Supplementary Fig. 35 | NH_3 yield rate for Pd-Cl/Cu_2O under different reaction conditions.

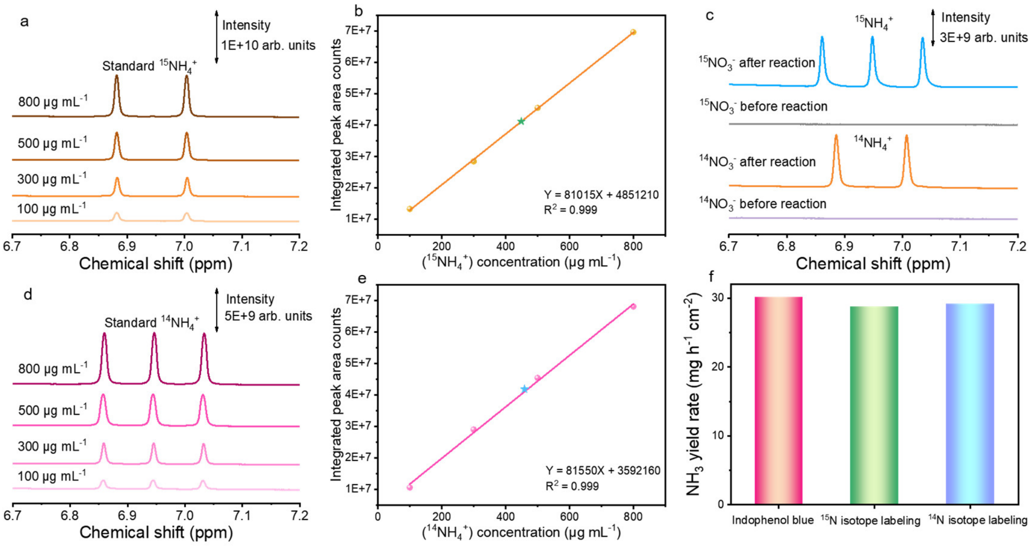

Supplementary Fig. 36 | ^{1}H NMR spectra for (a) $^{15}NH_4Cl$ and (d) $^{14}NH_4Cl$ standard solutions, respectively. Calibration curves for (b) $^{15}NH_4Cl$ and (e) $^{14}NH_4Cl$ standard solutions, respectively. The green stars represented the concentrations of produced $^{15}NH_4Cl$ after $^{15}NO_3^-$ electrolysis for 1 h. The blue stars represented the concentrations of produced $^{14}NH_4Cl$ after $^{14}NO_3^-$ electrolysis for 1 h. (c) ^{1}H NMR analysis of the electrolyte fed by $^{15}NO_3^-$ and $^{14}NO_3^-$ in NO_3RR. (f) The concentration of NH_4^+ measured by indophenol blue, and isotope labeling method. Reaction conditions: catalyst: Pd-Cl/Cu_2O; potential: -0.4 V vs. RHE.

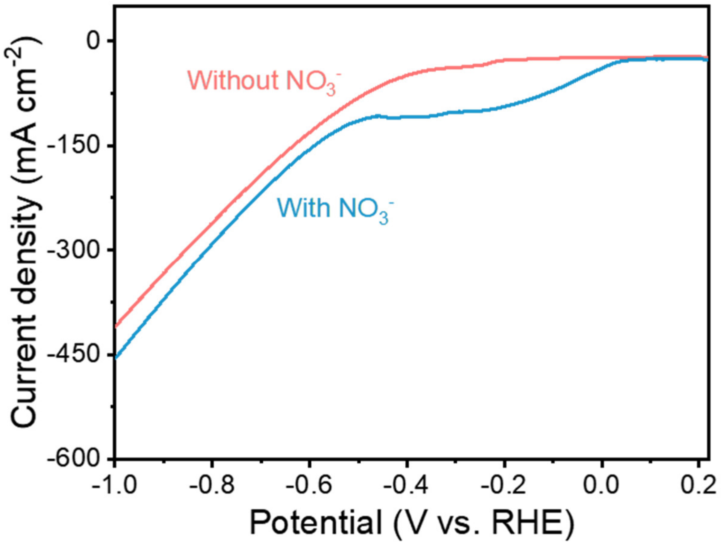

Supplementary Fig. 37 | LSV curves for Pd-Cl/Cu_2O in 1 M KOH with and without NO_3^-.

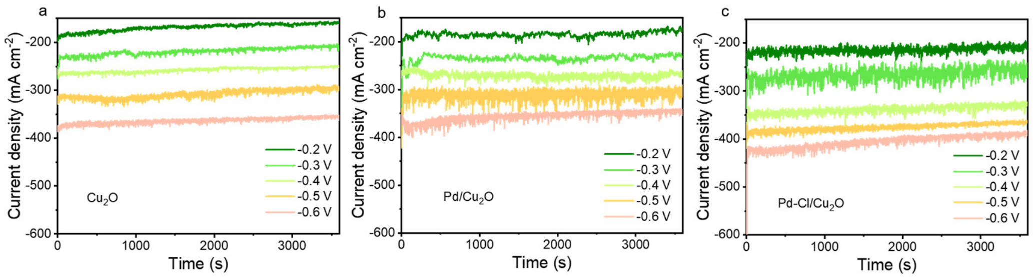

Supplementary Fig. 38 | Time-dependent current density curves of (a) Cu_2O, (b) Pd/Cu_2O, and (c) Pd-Cl/Cu_2O catalysts at various potentials for 1 h electrolysis.

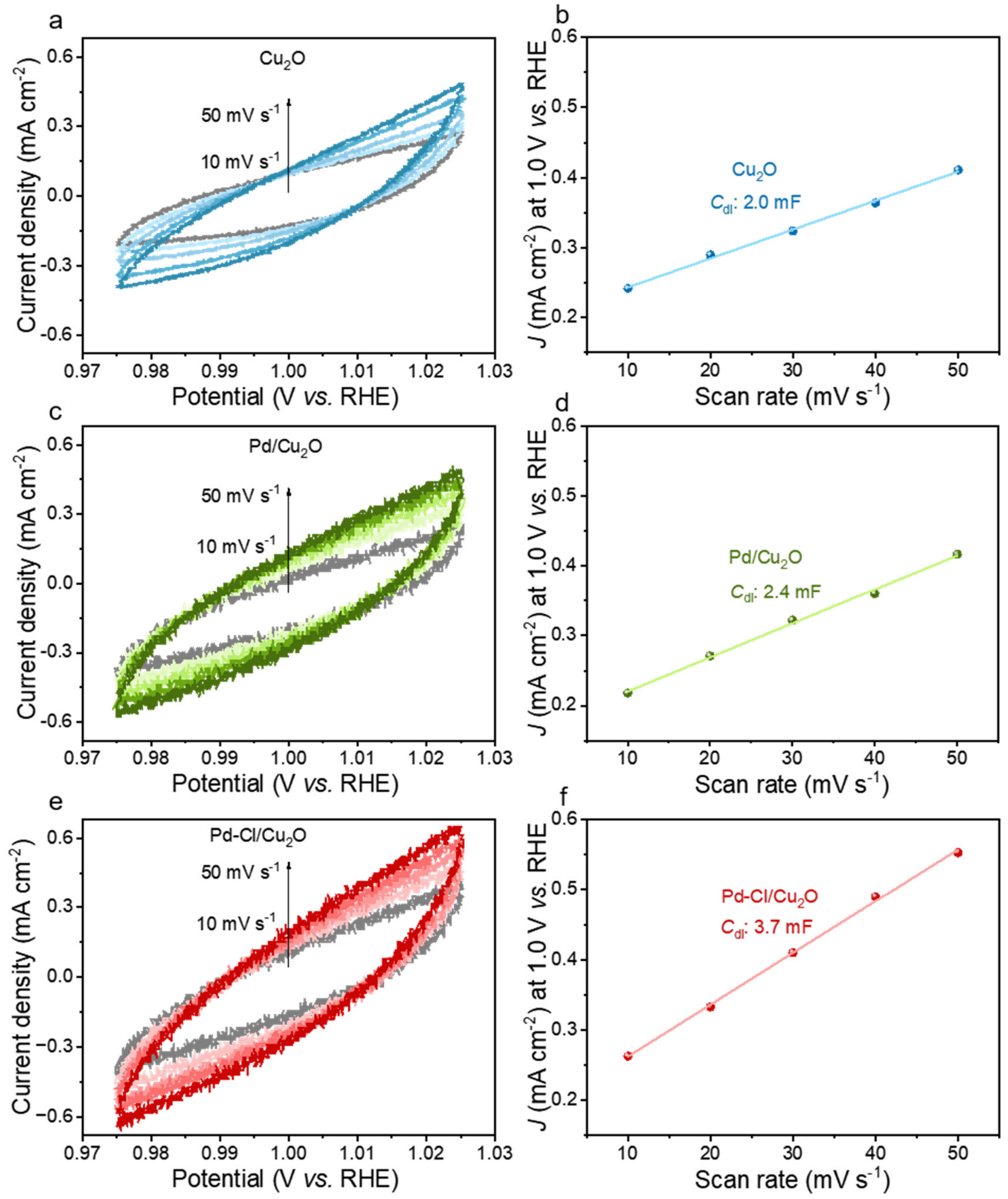

Supplementary Fig. 39 | (a, c, e) Cyclic voltammetry curves of catalysts at different scan rates. (b, d, f) Current density differences at 1.0 V vs. RHE against scan rates to calculate C_{dl}.

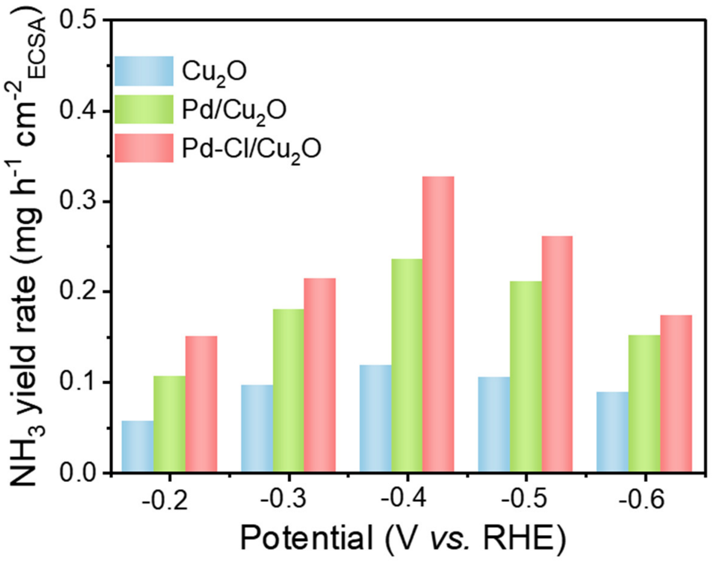

Supplementary Fig. 40 | The electrochemical-surface-area-normalized NH_3 yields rate at different applied potentials on catalysts.

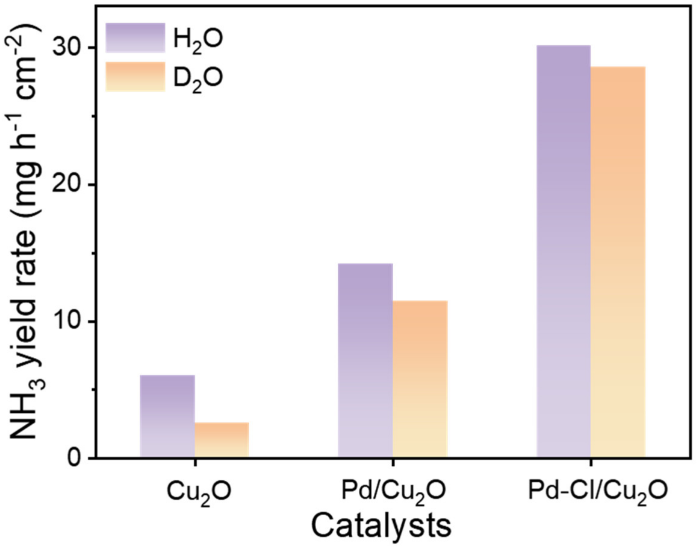

Supplementary Fig. 41 | NH_3 yield rate of catalysts measured in a 1 M KOH with 56 mM NO_3^- electrolyte at -0.4 V vs. RHE.

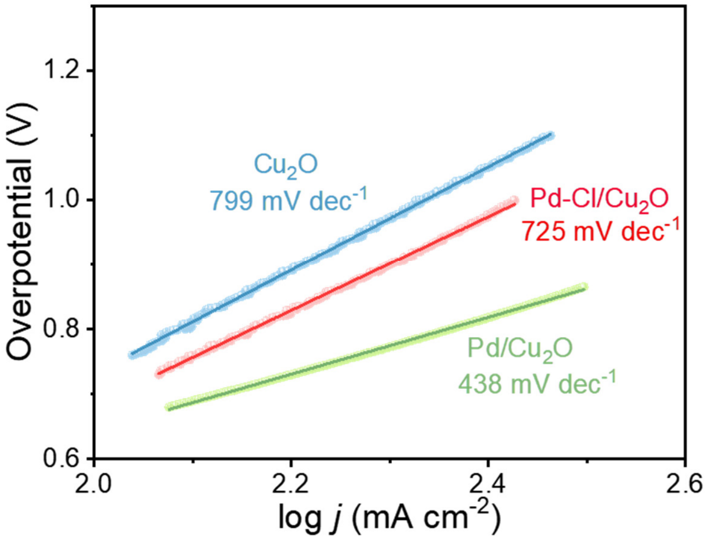

Supplementary Fig. 42 | Tafel curves of catalysts.

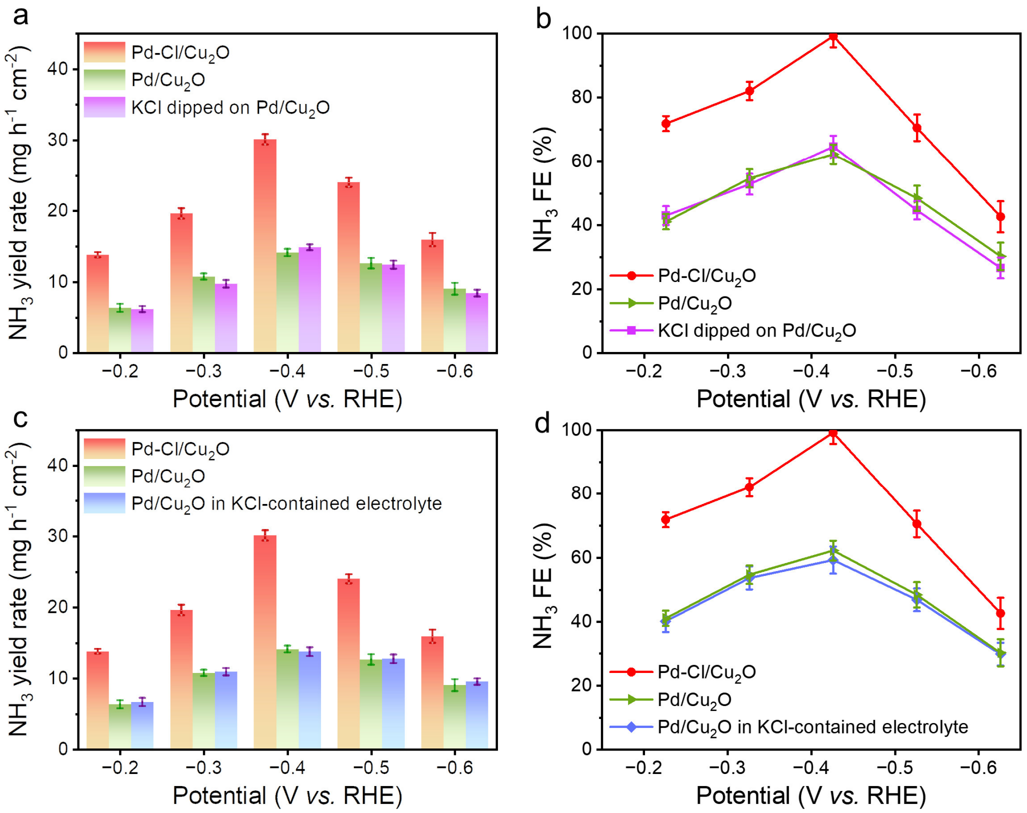

Supplementary Fig. 43 | (a, c) NH_3 yield rate and (b, d) NH_3 FE of various catalysts in a 1 M KOH with 56 mM NO_3^- electrolyte for 1 h electrolysis.

Notes: To elucidate the source of Cl that induces enhanced NH_3 production activity, we supplemented two groups of control experiments. The first group is to dip a certain amount of KCl on Pd/Cu_2O electrode, while the second group is to add KCl to the reaction electrolyte in Pd/Cu_2O systems for testing NO_3RR performance. As shown in Supplementary Fig. 43, the NH_3 yield rate and NH_3 FE of the two control experiments are comparable to the Pd/Cu_2O, and far lower than that of $Pd/Cl-Cu_2O$. This result proves that introducing Cl species in the sample synthesis to prepare Cl coordinated Pd single atom catalysts can effectively achieve impressive NO_3RR performance, through Cl ligand regulating the electronic structure of Pd atoms to mediate the proton feeding. While simply adding Cl ions to the reaction system would not significantly improve the NO_3RR performance. Hence, the Cl effect arises from the synthesis.

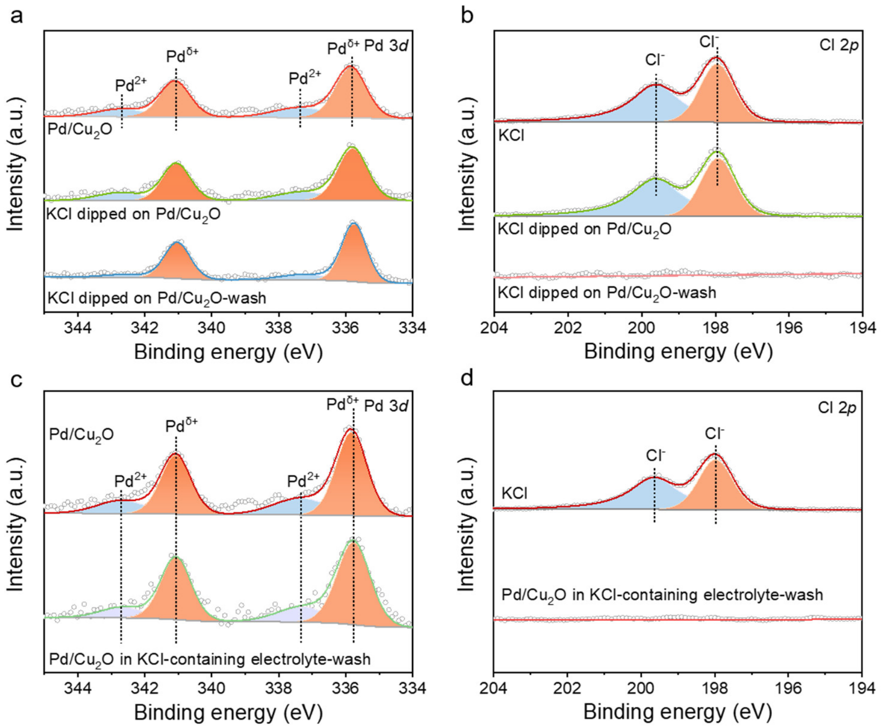

Supplementary Fig. 44 | (a, c) Pd 3*d* and (b, d) Cl 2*p* XPS spectra of samples.

Notes: (1) In the synthesis process of Pd-Cl/Cu_2O, chloride was utilized as the precursor to bind and stabilize the Pd atoms by impregnation and hydrogen reduction approach. During the low-temperature treatment procedure, the Cl ligand-mediated effect triggers the formation of Cl coordinated with Pd atom on Cu_2O matrix. According to the quantitative least-squares best-fitting of EXAFS spectra (Fig. 3f and Supplementary Table 1) and XPS spectra (Supplementary Figs. 21c-d and 22), there exists a strong interaction between two Cl and one Pd single atom in the as-prepared Pd-Cl/Cu_2O catalyst. After electrolysis, the unchanged EXAFS results further confirm the stable structure of Cl coordinated Pd (Supplementary Fig. 52c and Supplementary Table 3). Relevant literatures (*Nat. Catal.*, **3**, 376-385 (2020), *Nat. Commun.*, **13**, 6875 (2022); *Electrochem. Energ. Rev.*, **2**, 539-573 (2019).) have reported that Cl ligand can stable single atoms (Au, Pt, and Ru etc.).

(2) The control experiments suggest that simply dipping Cl^- on the Pd/Cu_2O or adding Cl^- in the electrolyte lead to weak or negligible interaction between Cl and Pd (Supplementary Fig. 44). The Cl^- can easily be eliminated by washing the catalysts with deionized water, due to the weak bond of Pd-Cl. Thus, the two counterparts show comparable NO_3RR activity to pure Pd/Cu_2O, and far lower than that of Pd-Cl/Cu_2O (Supplementary Fig. 43).

(3) In summary, the Cl acts as a synthetic directing agent to stabilize Pd single atoms, which could form strong Pd-Cl bond. Meanwhile, two Cl coordinated with one Pd single atom is the most stable structure.

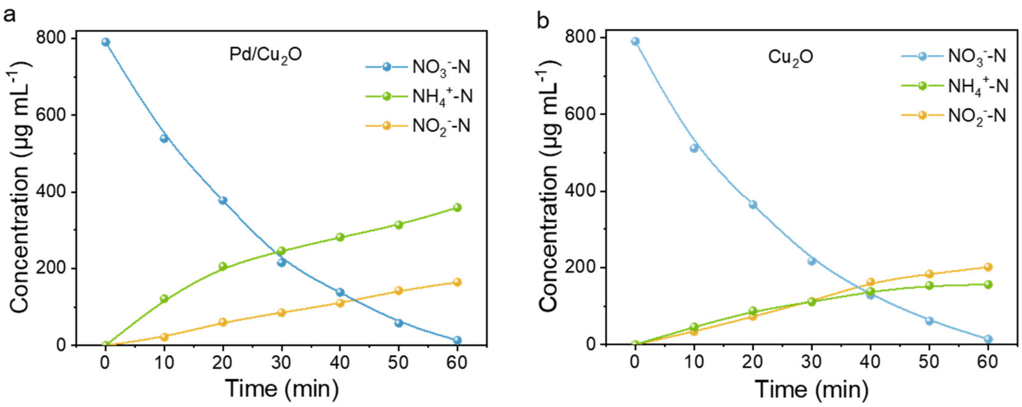

Supplementary Fig. 45 | NO_3^- removal over (a) Pd/Cu_2O and (b) Cu_2O catalysts at -0.4 V vs. RHE in 1 M KOH + 56 mM NO_3^- electrolyte (equals 790.3 μg mL^{-1} NO_3^-–N).

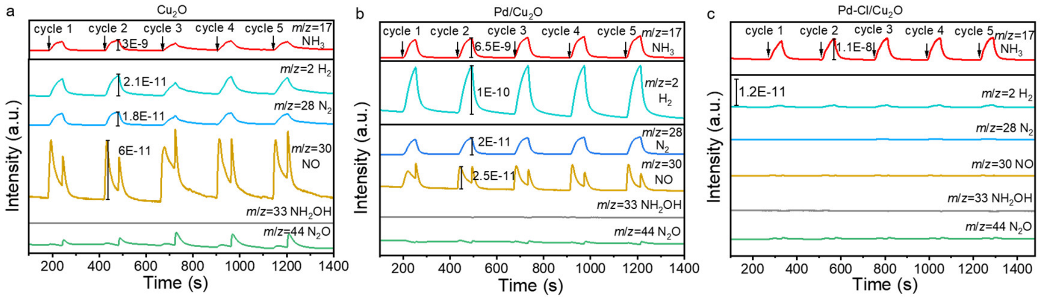

Supplementary Fig. 46 | Online differential electrochemical mass spectrometry (DEMS) measurements of NO_3RR over (a) Cu_2O, (b) Pd-Cu_2O, and (c) Pd-Cl/Cu_2O under the potential of -0.4 V vs. RHE.

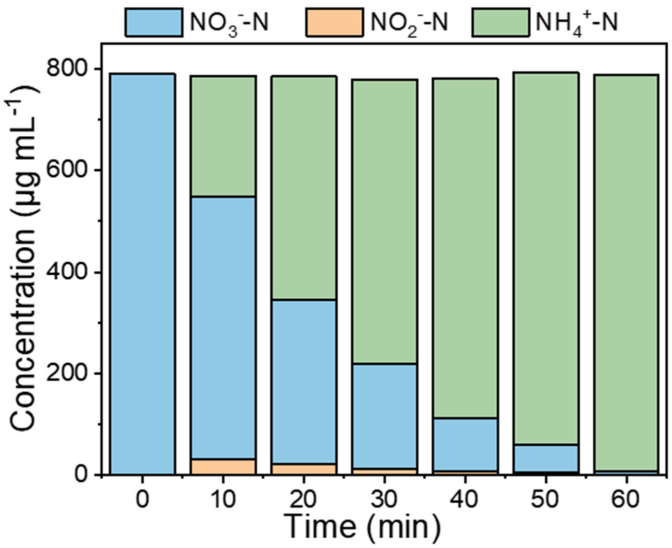

Supplementary Fig. 47 | Product concentrations at different times using Pd-Cl/Cu_2O catalysts in 1 M KOH with 56 mM NO_3^- electrolytes during NO_3RR.

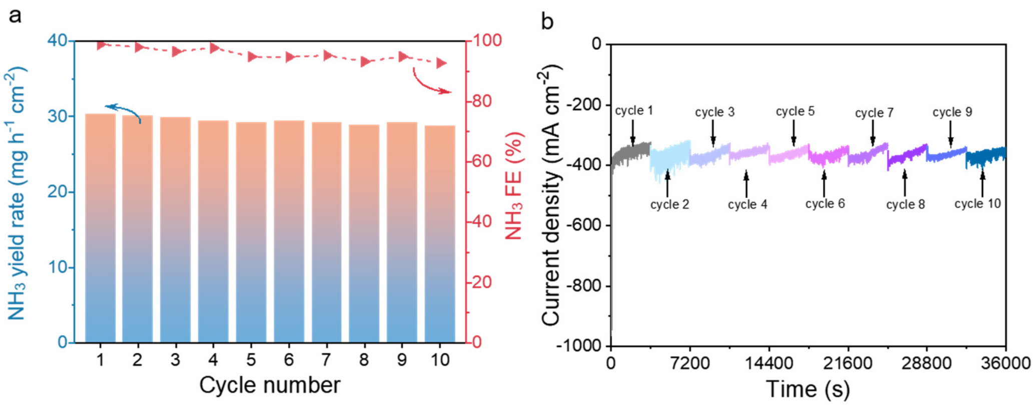

Supplementary Fig. 48 | (a) NO_3RR performance stability over Pd-Cl/Cu_2O measured in a 1 M KOH with 56 mM NO_3^- electrolyte at -0.4 V vs. RHE. (b) Time-dependent current density curves of NO_3RR over Pd-Cl/Cu_2O for successive 10 cycles.

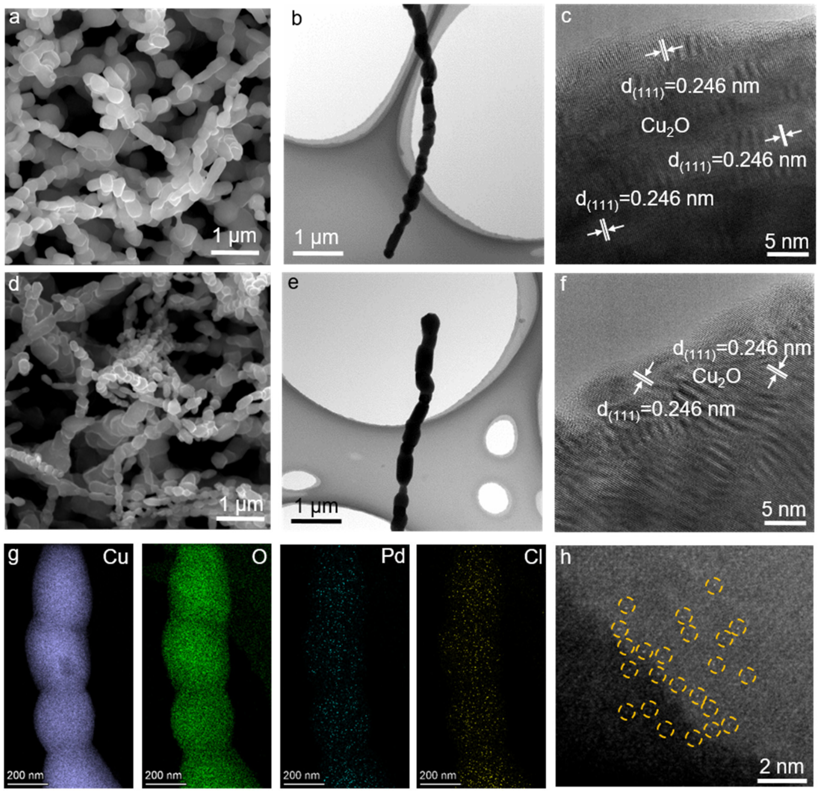

Supplementary Fig. 49 | (a) SEM, (b) TEM, and (c) HRTEM images of Pd-Cl/Cu_2O before NO_3RR. (d) SEM, (e) TEM, (f) HRTEM images, (g) EDX mapping, and (h) AC-HAADF-STEM image of Pd-Cl/Cu_2O after NO_3RR.

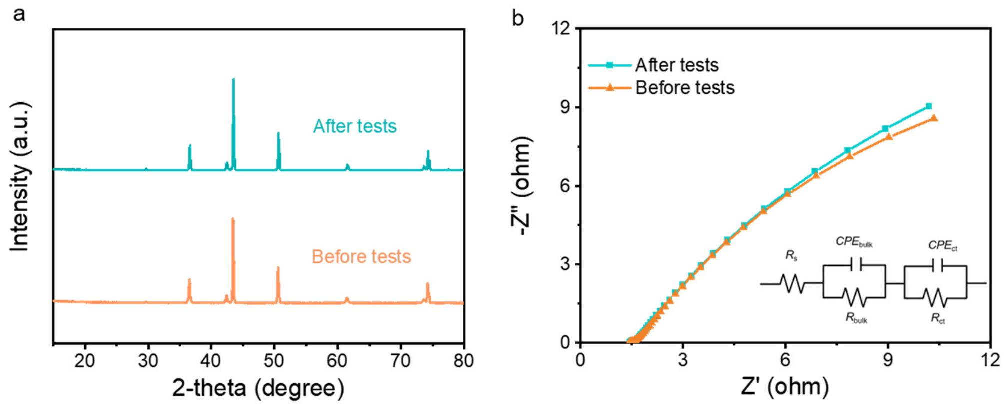

Supplementary Fig. 50 | (a) XRD patterns and (b) Nyquist plots of Pd-Cl/Cu_2O before and after NO_3RR.

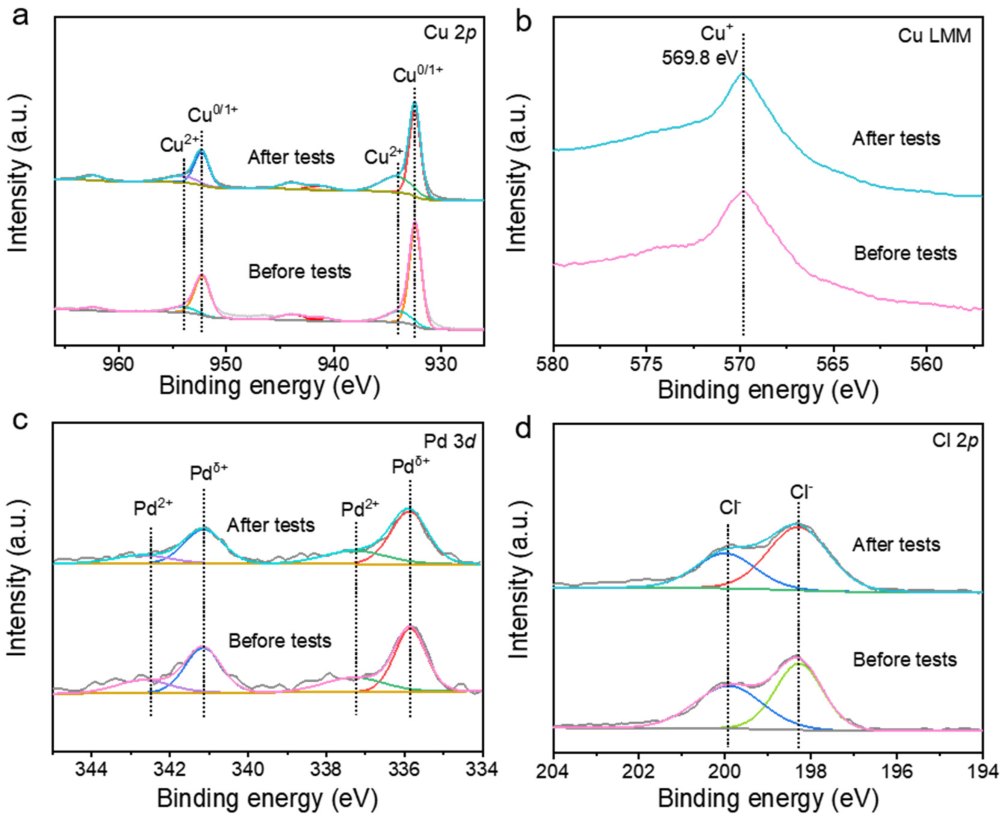

Supplementary Fig. 51 | (a) Cu 2*p* XPS spectra, (b) Cu LMM Auger spectra, (c) Pd 3*d* XPS spectra, and (d) Cl 2*p* XPS spectra of Pd-Cl/Cu_2O before and after NO_3RR.

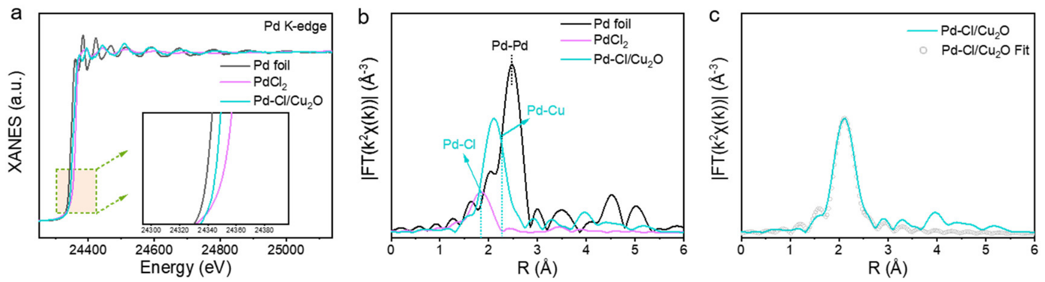

Supplementary Fig. 52 | (a) Pd K-edge XANES spectra, (b) FT k^2-weighted, and (c) fitting EXAFS spectra of Pd-Cl/Cu_2O after NO_3RR.

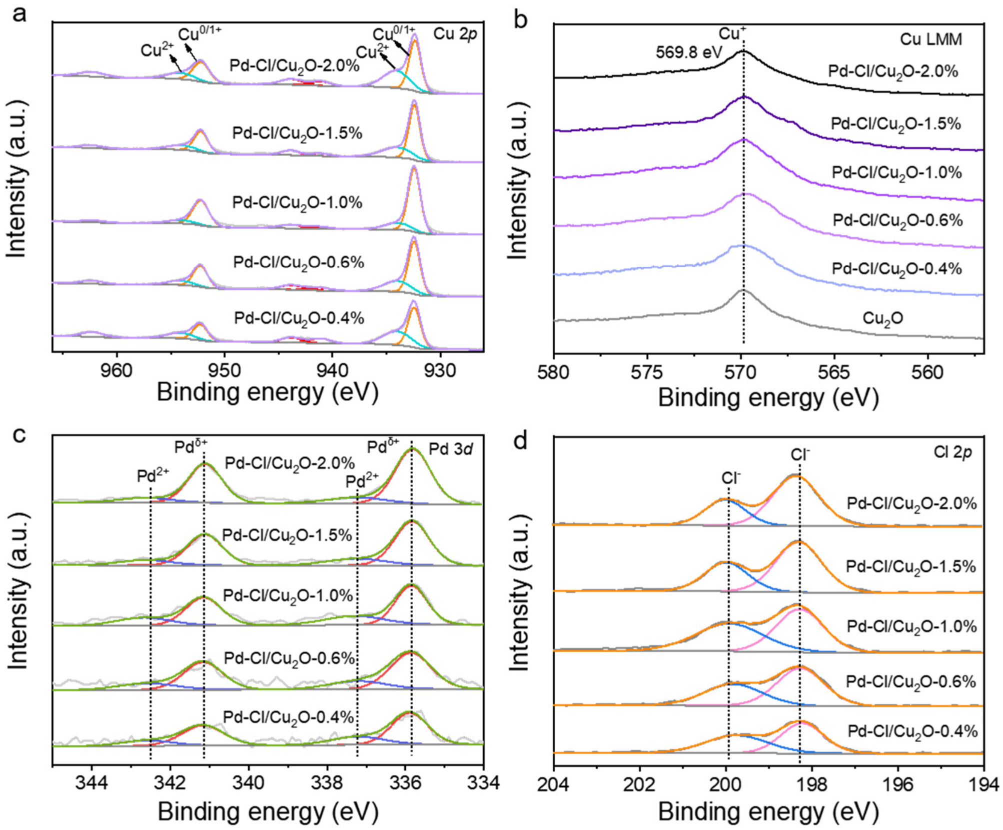

Supplementary Fig. 53 | (a) Cu 2*p* XPS spectra, (b) Cu LMM Auger spectra, (c) Pd 3*d* XPS spectra, and (d) Cl 2*p* XPS spectra of catalysts.

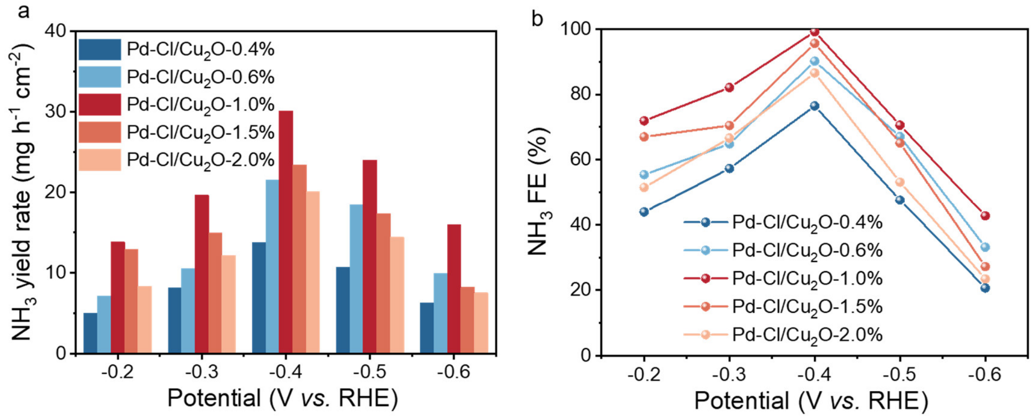

Supplementary Fig. 54 | (a) NH_3 yield rate and (b) NH_3 FE of catalysts in a 1 M KOH with 56 mM NO_3^- electrolyte.

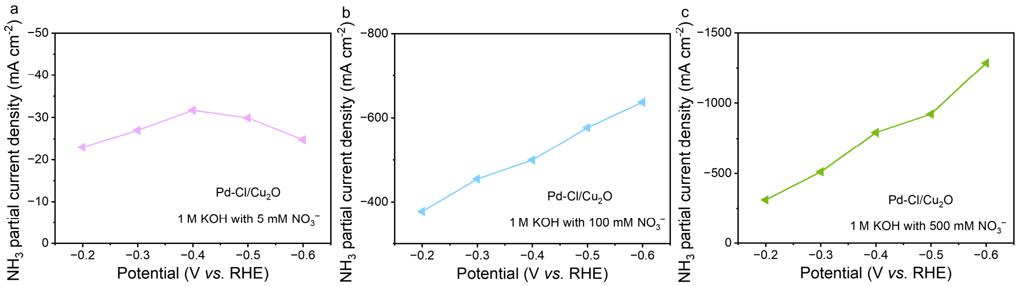

Supplementary Fig. 55 | NH_3 partial current densities of Pd-Cl/Cu_2O in a 1 M KOH electrolyte with (a) 5 mM, (b) 100 mM, and (c) 500 mM NO_3^- concentrations under potential range from -0.2 to -0.6 V vs. RHE.

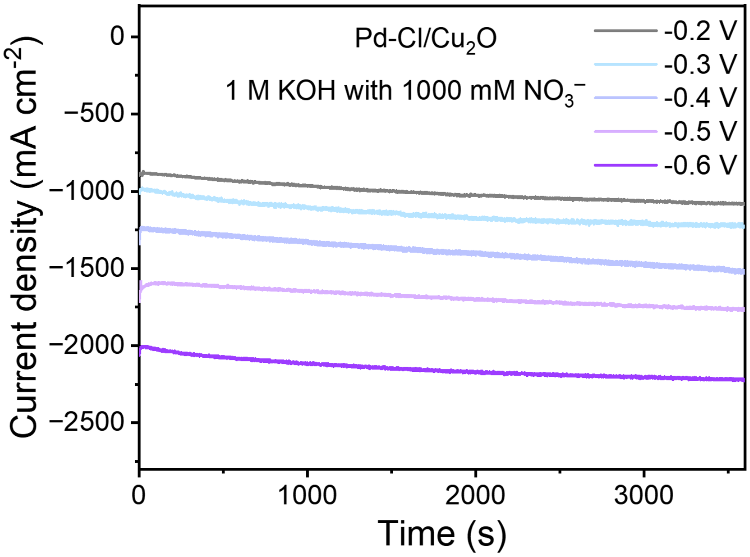

Supplementary Fig. 56 | Current density of Pd-Cl/Cu_2O in a 1 M KOH electrolyte with 1000 mM NO_3^- under potential range from -0.2 to -0.6 V vs. RHE.

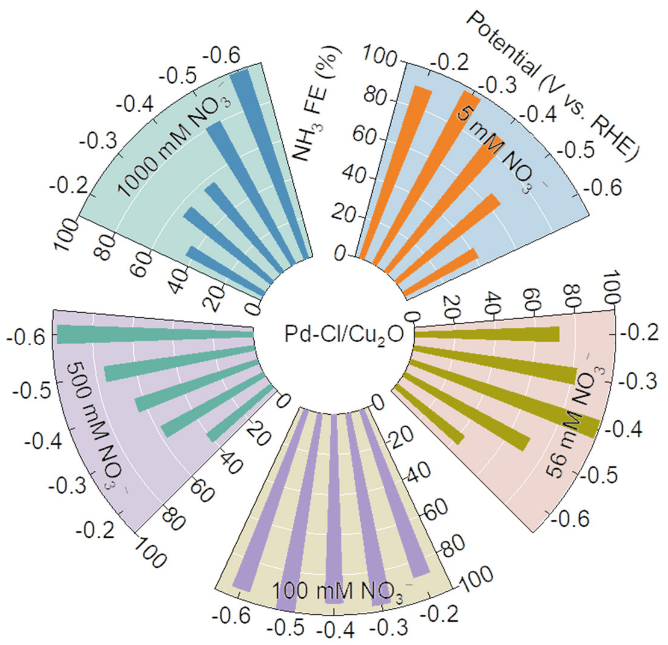

Supplementary Fig. 57 | NH_3 FE of Pd-Cl/Cu_2O in a 1 M KOH electrolyte with different NO_3^- concentrations.

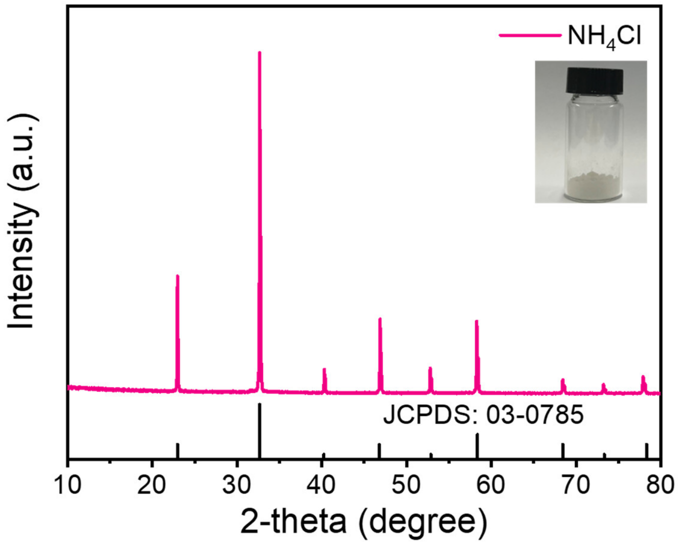

Supplementary Fig. 58 | Synthesized NH_4Cl product and its XRD pattern. Inset: the product itself.

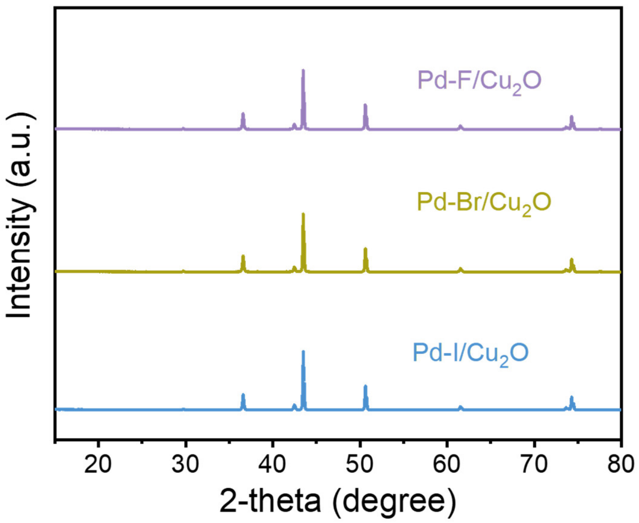

Supplementary Fig. 59 | XRD patterns of catalysts.

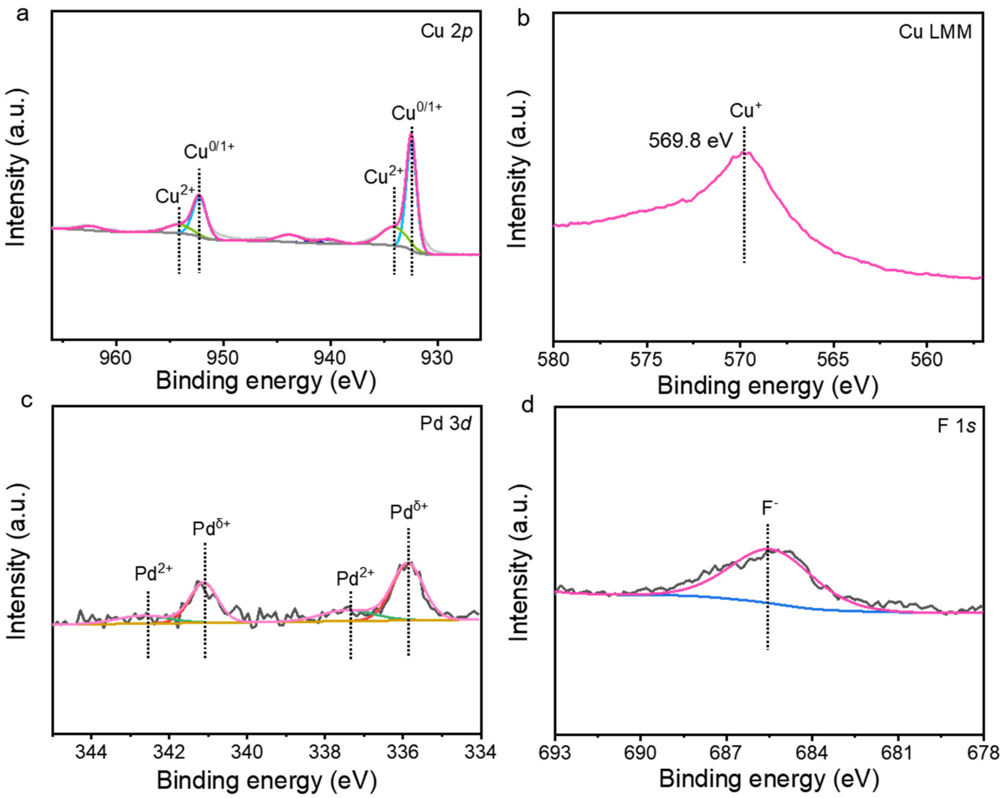

Supplementary Fig. 60 | (a) Cu 2*p* XPS spectra, (b) Cu LMM Auger spectra, (c) Pd 3*d*, and (d) F 1*s* XPS spectra of Pd-F/Cu_2O.

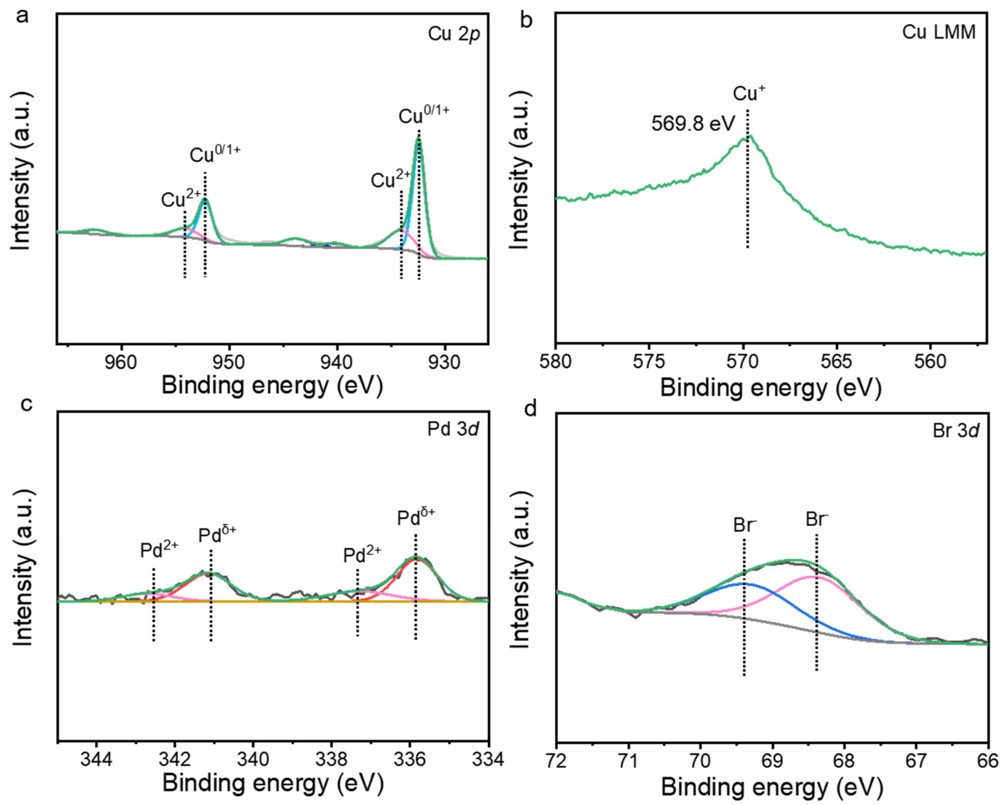

Supplementary Fig. 61 | (a) Cu 2*p* XPS spectra, (b) Cu LMM Auger spectra, (c) Pd 3*d*, and (d) Br 3*d* XPS spectra of Pd-Br/Cu_2O.

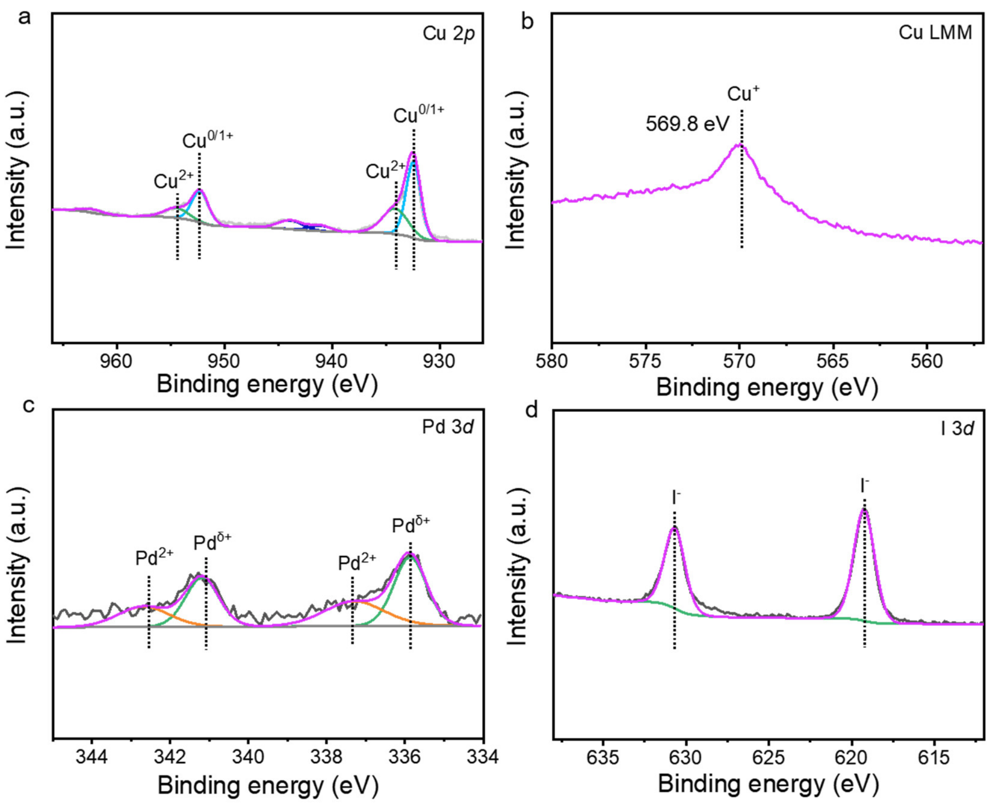

Supplementary Fig. 62 | (a) Cu 2*p* XPS spectra, (b) Cu LMM Auger spectra, (c) Pd 3*d*, and (d) I 3*d* XPS spectra of Pd-I/Cu_2O.

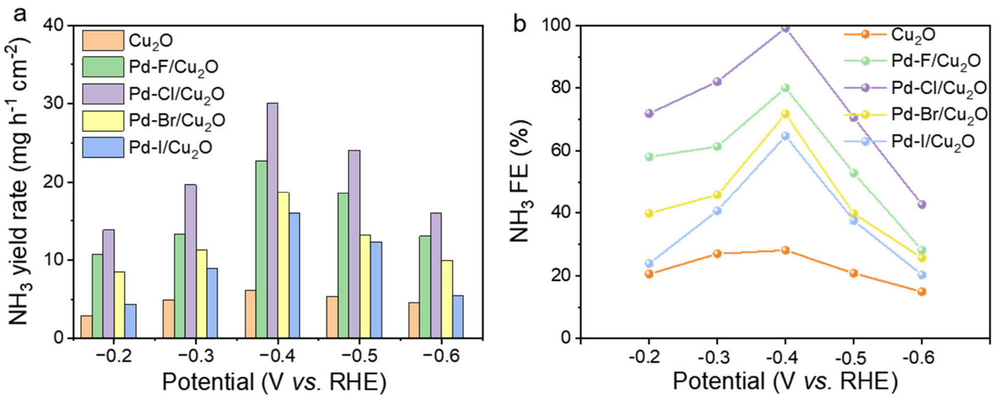

Supplementary Fig. 63 | (a) NH_3 yield rate and (b) NH_3 FE of catalysts in a 1 M KOH with 56 mM NO_3^- electrolyte.

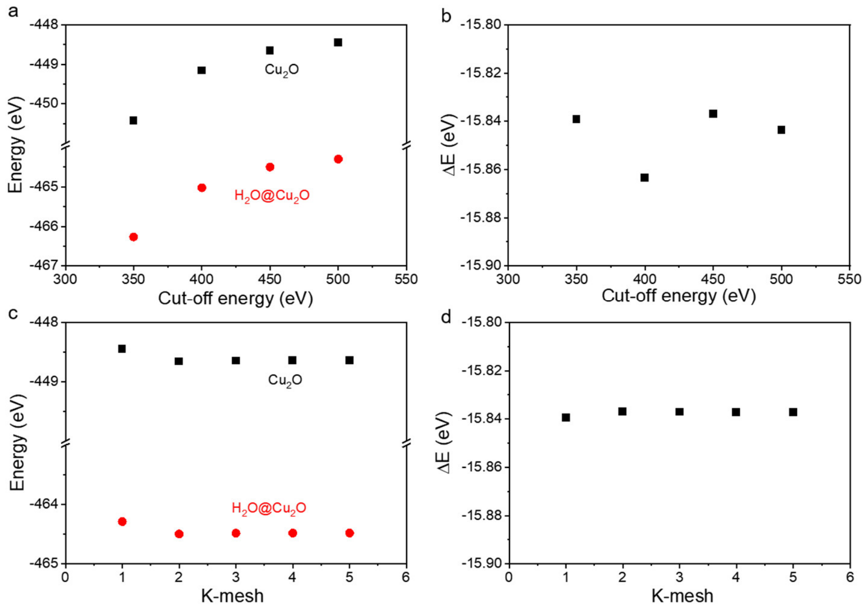

Supplementary Fig. 64 | The energy (**a**) and energy difference (**b**) on Cu_2O model before and after H_2O adsorption under various cut-off energies. The energy (**c**) and energy difference (**d**) on Cu_2O model before and after H_2O adsorption under different K-points.

Notes: We have calculated the various cut-off energies (from 350 to 500 eV) and different K points (from $1 \times 1 \times 1$ to $5 \times 5 \times 1$) on Cu_2O model before and after H_2O adsorption to investigate their impact on the energy of the entire reaction system. As shown in Supplementary Figs. 63a-b, under a series of cut-off energies, the energy difference between the Cu_2O model before and after H_2O adsorption is relatively small. Similarly, the energy change on the models under different K-points is also not significant (Supplementary Figs. 63c-d). Therefore, we chose the $2 \times 2 \times 1$ K-point mesh and 450 eV cut-off energy for calculation.

Supplementary Table 1 | The Pd K-edge EXAFS fitting results for Pd-Cl/Cu_2O.

Sample	Shell	CN	R(Å)	σ^2(Å^2)	ΔE_0(eV)	R factor
Pd-Cl/Cu_2O	Pd-Cl	1.964	2.34	0.007	22.7	0.0008
	Pd-Cu	3.004	2.52	0.002	7.80	

Supplementary Table 2 | The Pd K-edge EXAFS fitting results for Pd/Cu_2O.

Sample	Shell	CN	R(Å)	σ^2(Å^2)	ΔE_0(eV)	R factor
Pd/Cu_2O	Pd-Cu	3.04	2.62	0.0008	-3.09	0.007

Supplementary Table 3 | The Pd K-edge EXAFS fitting results for Pd-Cl/Cu_2O after NO_3RR.

Sample	Shell	CN	R(Å)	σ^2(Å^2)	ΔE_0(eV)	R factor
Pd-Cl/Cu_2O	Pd-Cl	1.961	2.33	0.0005	20.75	0.0004
	Pd-Cu	3.005	2.58	0.0006	1.68	

Supplementary Table 4 | Fitting results for resistances of Pd-Cl/Cu_2O before and after NO_3RR tests according to EIS equivalent circuit diagram.

Resistance / Sample tests	R_s (Ω)	R_{bulk} (Ω cm^{-2})	R_{ct} (Ω cm^{-2})
Before tests	1.45	0.156	44.3
After tests	1.46	0.175	48.7

Note: R_s is the external circuit resistance. R_{bulk} is the bulk trapping resistance. R_{ct} is the interfacial charge transfer resistance.

Supplementary Table 5 | The content of Pd elements for the series Pd-Cl/Cu_2O catalysts determined by ICP-OES.

Sample	Pd (wt.%)
Pd-Cl/Cu_2O-0.4%	0.44
Pd-Cl/Cu_2O-0.6%	0.61
Pd-Cl/Cu_2O-1.0%	0.98
Pd-Cl/Cu_2O-1.5%	1.55
Pd-Cl/Cu_2O-2.0%	1.98

Supplementary Table 6 | Comparison of NO_3RR performance of Pd-Cl/Cu_2O with reported works.

Catalyst	Electrolyte	NH_3 FE (%)	NH_3 production rate (mg h^{-1} cm^{-2})	NH_3 partial current (mA cm^{-2})	Ref.
**Pd-Cl/Cu_2O**	**56 mM KNO_3 + 1 M KOH**	**99.2**	**30.1**	**~350**	**This work**
**Pd-Cl/Cu_2O**	**1000 mM KNO_3 + 1 M KOH**	**99.1**	**~330**	**~2180**	**This work**
$Ru_{15}Co_{85}$HNDs	1000 mM KNO_3 + 1 M KOH	97	~119	~1000	Nat. Cat. 2023, 6, 404-412
Ru-CuNW	32 mM KNO_3 + 1 M KOH	96	76.6	965	Nat. Nanotechnol. 2022, 17, 759-767
Cu-PTCDA	8.1 mM KNO_3 + 1 M PBS	85.9	0.44	-	Nat. Energy 2020, 5, 605-613
Electroreduction $Co(OH)_2$ nanoarrays	100 mM KNO_3 + 1 M KOH	98	176.8	~2156	Adv. Sci. 2021, 8, 2004523
$Cu_{50}Co_{50}$ nanosheet	100 mM KNO_3 + 1 M KOH	100	81.6	1035	Nat. Commun. 2022, 13, 7899
Fe single-atom catalyst (SAC)	500 mM KNO_3 + 0.1 M K_2SO_4	75	7.8	30	Nat. Commun. 2021, 12, 2870
Fe-PPy SAC	100 mM KNO_3 + 0.1 M KOH	~100	2.8	34.6	Energy Environ. Sci. 2021, 14, 3522-3531
Pd	20 mM $NaNO_3$ + 0.1 M NaOH	35	0.34	4.25	ACS Catal. 2021, 11, 12, 7568-7577
Ru nanoclusters	1000 mM KNO_3 + 0.1 M KOH	~100	17.5	251	J. Am. Chem. Soc. 2020, 142, 7036−7046
Cu/Cu_2O NWAs	14.3 mM KNO_3 + 0.5 M Na_2SO_4	95.8	4.2	52.5	Angew. Chem. Int. Ed. 2020, 59, 5350-5354
$Cu_{50}Ni_{50}$ alloy	100 mM KNO_3 + 1 M KOH	99	7.1	90	J. Am. Chem. Soc. 2020, 142, 5702−5708
CuPd	1000 mM KNO_3 + 1 M KOH	92.5	18.1	240	Nat. Commun. 2022, 13, 2338
Rh@Cu	100 mM KNO_3 + 0.1 M Na_2SO_4	93	13.6	162	Angew. Chem. Int. Ed. 2022, 61, e202202556
FTO-E	100 mM $NaNO_3$+ 0.1 M PBS	87.6	1.2	-	Angew. Chem. Int. Ed. 2023, 62, e202215782
Cu@C	1 mM KNO_3 + 1 M KOH	72	0.47	-	Adv. Mater. 2022, 34, 2204306

Cu/Cu_2O nanowires	3.2 mM $NaNO_3$ + 0.5 M Na_2SO_4	81.2	4.1	-	Angew. Chem. Int. Ed. 2020, 59, 5350-5354
a-RuO_2	3.2 mM $NaNO_3$ + 0.5 M Na_2SO_4	97.46	2.0		Angew. Chem. Int. Ed. 2022, 134, e202202604
Poly-Cu_{14}cba	4 mM KNO_3 + 0.5 M K_2SO_4	90	2.8		Angew. Chem., Int. Ed. 2022, 134, e202114538
Ru_1Cu_{10}/rGO 1 wt.% loading	100 mM KNO_3 + 1 M KOH	63	1.3	37	Adv. Mater. 2023, 35, 2202952
O-SiNW/Au	10 mM HNO_3 + 0.5 M K_2SO_4	95.6	4.4		Angew. Chem., Int. Ed. 2022, 61, e202204117
Fe/Ni_2P	50 mM KNO_3 + 0.2 M K_2SO_4	94.3	4.2		Adv. Energy Mater. 2022, 12, 2103872
